# Urinary Metabolome Study for Monitoring Prostate Cancer Recurrence Following Radical Prostatectomy

**DOI:** 10.3390/cancers17172756

**Published:** 2025-08-24

**Authors:** Sabur Badmos, Elizabeth Noriega Landa, Kiana L. Holbrook, George E. Quaye, Xiaogang Su, Wen-Yee Lee

**Affiliations:** 1 Department of Chemistry and Biochemistry, University of Texas at El Paso, El Paso, TX 79968, USAenoriegalanda2@utep.edu (E.N.L.); kholbrook@miners.utep.edu (K.L.H.); 2 Division of Health Services and Outcomes Research, Children’s Mercy Kansas City, Kansas City, MO 64108, USA; gequaye@cmh.edu; 3 Department of Mathematical Sciences, University of Texas at El Paso, El Paso, TX 79968, USA; xsu@utep.edu

**Keywords:** prostate cancer diagnosis, radical prostatectomy, GC-MS, volatile organic compounds, VOCs, PLS-DA, biochemical recurrence, metastatic recurrence, urinary biomarkers

## Abstract

Background/objectives: Prostate cancer (PCa) is the most common cancer among males. Approximately 20–40% of patients with clinically localized PCa will present with a biochemical recurrence after a radical prostatectomy (RP), while some will present with recurrent metastasis. Monitoring the disease post-treatment is crucial for detecting a potential cancer recurrence early. Urinary volatile organic compounds (VOCs) have shown potential to detect PCa. However, their application in disease monitoring remains unexplored. Methods: A total of 165 urine samples were collected from male adults with biopsy-designated PCa-positive results before (*n* = 55) and after a RP (*n* = 55), and with biopsy-designated PCa-negative diagnosis (*n* = 55). The post-RP cohort was subdivided into three groups based on their health status after surgery as recovered healthy, biochemical recurrence, and recurrent metastasis. VOCs in the urine samples were extracted by stir bar sorptive extraction and analyzed using gas chromatography and mass spectrometry. We explored the use of metabolomics and a machine learning algorithm tool to investigate the potential of using VOCs for differentiating PCa diagnoses before and after the RP procedure with different outcomes. Results: Over 100 potential VOCs were identified to differentiate PCa patients before and after a RP, and those with biochemical recurrence and recurrent metastasis. Conclusions: Urinary VOCs are promising biomarkers that could be used to differentiate PCa patients pre- and post-RP. The findings from this research provide preliminary insights and could aid future investigations in developing tools for PCa patients after treatment. The absence of a validation cohort limits the reproducibility and translational impact of these findings; therefore, the results should be considered exploratory and require confirmation in larger, independent cohorts.

## Simple Summary

Approximately 20–40% of patients with localized prostate cancer (PCa) will present with a biochemical recurrence (BCR) after radical prostatectomy (RP), while some will present with recurrent metastasis (RCM). The focus of the research was to investigate the application of urinary metabolites in PCa monitoring post-RP. Urine samples from the patients with PCa-positive results before and after a RP, and from patients without PCa, were obtained. Over 100 urinary metabolites were found to be significantly different between the pre- and post-RP groups and between the BCR and RCM groups. Findings from this research could aid in the development of rapid tools for patients after a RP for detecting potential cancer recurrence early.

## 1. Introduction

Prostate cancer (PCa) is the most prevalent and non-cutaneous cancer among males, and it is the second leading cause of cancer-related deaths among men in the United States [1]. There are various methods of treating and managing this disease, such as observation or active surveillance, radical prostatectomy, radiation therapy, cryotherapy, hormone therapy, chemotherapy, immunotherapy, targeted-drug therapy, high-intensity focused ultrasound, and other ablative treatments for prostate cancer, such as photodynamic therapy and focal laser ablation [2,3,4,5,6,7,8]. The treatment plans largely depend on a variety of factors, including the overall health and well-being of the patient, the patient’s age and Gleason score grade, the associated risks of cancer, the nature of the tumor, and the goals for the treatment outcomes [9,10,11]. Additionally, ongoing efforts are to develop beneficial PCa screening methods to mitigate the harms and challenges that are associated with overdiagnosis and overtreatment via the use of molecular biomarkers and magnetic resonance imaging-targeted biopsy [9,10,11,12,13]. These recent advances in medical diagnostic technology for early detection and rapid treatment have resulted in the number of cancer survivors continuing to increase in the United States [14,15].

Radical prostatectomy (RP) remains the primary treatment for localized PCa and has been performed for many years, with excellent oncologic control [16,17,18]. Nevertheless, approximately 20–40% of patients with clinically localized PCa will present with a biochemical recurrence (BCR) within 10 years after their initial definitive therapy, while some will be diagnosed with recurrent metastasis after a RP [19,20,21]. These patients have elevated prostate-specific antigen (PSA) levels that indicate the disease has returned; however, imaging examinations do not show the presence of cancer. Furthermore, some patients will present with recurrent metastasis, meaning that the cancer has spread to a distant place in the body [14,15,22,23,24]. Over time, it has been observed that PSA relapse presents several outcomes according to clinicopathological features, such as the Gleason score, PSA doubling time, clinical stage, and surgical margin status [24,25,26]. Despite these challenges, PSA remains the main tool used for assessing disease progression after a RP.

Metabolomics has been explored for cancer diagnosis and monitoring [27,28], as it assesses numerous small biomolecules, such as amino acids, nucleotides, sugars, and lipids, which are products and intermediate molecules of the metabolic pathways [29,30,31]. These metabolites can serve as biomarkers, providing vital information for diagnosis, prognosis, and treatment of diseases [32,33,34,35]. Among them, volatile organic compounds (VOCs) have gained attention for cancer diagnosis [36,37]. VOCs, which are the end-products of cell metabolism, can be detected in bodily fluids like urine, breath, sweat, and blood [37,38]. Their identities and concentrations reflect an individual’s metabolic state [39,40]. Research suggests that changes in the VOC profile may occur in patients with prostate cancer, offering a potential avenue for monitoring the patient’s disease status [41,42,43]. One advantage of using urinary VOC profiling is its non-invasiveness and abundance. Collecting urine samples is simple and convenient for patients when compared to blood drawing or imaging procedures. Additionally, VOC profiling techniques, such as gas chromatography–mass spectrometry (GC-MS) or electronic nose (e-nose) devices, can provide rapid results and may be cost-effective once established [38,44,45]. Thus, analyzing the changes in the components of VOC profiles could potentially provide important information regarding the molecular mechanisms behind a disease, the pathophysiological state, as well as presenting new approaches for personalized screening, monitoring, diagnosis, and prognosis.

The body of research exploring VOCs for early PCa diagnosis is continuously growing, with numerous investigations reporting potential volatile biomarkers of the disease. It was suggested that pathological conditions could affect or vary the VOC concentration in the human biological system, thereby leading to variations in the VOC concentrations present in the urine of subjects with physiological conditions when compared with patients without the conditions [46,47]. The use of VOCs in the diagnosis of PCa patients in comparison to healthy controls was reported [42,48,49]. The research on the diagnostic characteristics of VOCs in PCa is still in its early stages, and currently, VOCs are not widely explored as diagnostic tools. However, VOC analysis has the potential to offer a cost-effective and non-invasive way to diagnose PCa. VOC analysis may allow future disease stratification by providing insights into molecular alterations.

Consequently, effective clinical management of diseases, such as PCa, necessitates the identification of robust and reliable biomarkers with diagnostic and prognostic significance. Our recent studies have explored the use of VOCs in disease diagnosis and prognosis [41,42,50]. This study aimed to further explore the potential application of urinary VOCs that are associated with men who have undergone a radical prostatectomy yet continue to exhibit signs of the disease post-treatment. The urinary VOC profiles of prostate cancer patients, both pre- and post-radical prostatectomy, were analyzed to pinpoint potential metabolic signatures of PCa. This investigation aimed to provide an additional tool for clinicians in making decisions regarding the management of patients experiencing biochemical recurrence or recurrent metastasis of PCa. To the best of our knowledge, there are no directly comparable studies addressing this specific research question. This research hereby presents the first study that demonstrates the use of VOCs to investigate PCa biochemical and metastatic recurrence episodes in treated patients post-radical prostatectomy.

## 2. Materials and Methods

### 2.1. Chemicals and Materials

All the chemicals used were of analytical grade. Hydrochloric acid (37%) was obtained from Sigma-Aldrich (St. Louis, MO, USA). Ultra-pure deionized water (DI, Milli-Q benchtop Lab water purification system) (Millipore Inc., Burlington, MA, USA) was used to prepare the 2 M HCl solution and patients’ urine samples. Methanol (LC-MS grade, Burdick & Jackson, Muskegon, MI, USA) was used to prepare Mirex (internal standard, 99.0% purity; Dr. Ehrenstorfer GmbH, Augsburg, Germany) in a 100 mg/L solution. Stir bar sorptive extraction (SBSE) stir bars (Twister^®^, 10 mm × 1 mm) coated with polydimethylsiloxane and thermal desorption tubes (TDT) were obtained from GERSTEL (Mülheim, Germany).

### 2.2. Urine Samples Collection

The ethical approval (IRB 836503-9) for this study was obtained from the University of Texas at El Paso Internal Review Board Committee.

All urine specimens were purchased from the biorepository at the Macon & Joan Brock Virginia Health Sciences at Old Dominion University (Norfolk, VA, USA). The 110 paired urine samples used in this study were collected from male adults aged 45–80 years, who had biopsy-designated PCa-positive results pre- and post-radical prostatectomy (RP), and from 55 patients with a biopsy-designated PCa-negative assessment. The subjects were subdivided into three groups as follows: group 1—before a RP (*n* = 55); group 2—the post-RP (*n* = 55) group, and group 3—those who were biopsy-designated PCa-negative (*n* = 55), i.e., the healthy control. Furthermore, the post-RP group was subdivided into three groups based on their health status after surgery as follows: recovered healthy (RCH), biochemical recurrence (BCR), and recurrent metastasis (RCM). Details regarding the patients’ demographics are provided in Table 1.

### 2.3. Inclusion and Exclusion Criteria

In this study, sex is not considered a biological variable because PCa is a male-specific cancer. Exclusion was applied to patients who did not wish to participate in this study or whose urinalysis was suspicious of infection. A urine dipstick analysis was used on all patients to rule out any infection before an office-based transrectal ultrasound-guided biopsy was conducted. The patients’ urine samples (5 mL) were collected and stored at −80 °C before the urinary VOC analysis.

### 2.4. Volatile Organic Compounds Extraction from Urine Samples

To extract the VOCs from urine samples, the sample preparation protocol was developed in our group [41,42] was followed and is briefly stated as follows. The urine samples stored at −80 °C were thawed and centrifuged at 300× *g* for 10 min. Then, in a clean 20 mL amber vial, 1 mL of the supernatant was transferred, followed by the addition of 19 mL of deionized (DI) water, 600 µL of 2 M HCl, 300 µL of 1 ppm Mirex solution (internal standard), and a clean stir bar (TWISTER™, 10 mm × 1 mm, GERSTEL) was added. The mixture was stirred at 1000 rpm for 2 h. After stirring, the stir bar was removed, rinsed with DI water, dried with lint-free paper, and transferred into a thermal desorption tube (TDT). The TDTs were then placed in an autosampler mounted on a GC-MS for VOC analysis.

Sample randomization was applied prior to the GC-MS analysis to reduce potential batch effects. In addition, solvent blank samples were analyzed to account for compounds that were present in the reagents. The solvent blank samples for the urine samples consisted of 19.1 mL of HPLC-grade water, 300 µL of Mirex (1 ppm), 600 µL of HCl (2 M), and one stir bar (Twister, 10 mm × 1 mm). The VOCs found in the blank samples were used to account for endogenous compounds, specifically in column bleed, thermal desorption tubes, and stir bar emittances.

### 2.5. Gas Chromatography/Mass Spectrometry (GC-MS) Coupled with Thermal Desorption Unit

VOC analysis was conducted using an Agilent 8890 GC series system that was coupled with a mass spectrometer 5977B GC/MSD (Agilent Technologies, Wilmington, DE, USA). The instrumental settings for thermal desorption and GC-MS followed previously published methods [41,42]. The mass range explored was 20–500 *m*/*z*, and the data were generated using the scan mode, as previously reported. The Agilent Technologies GC-MS Enhanced Mass Hunter Workstation and Data Analysis Resource Application Software (MSD ChemStation G1701FA F.01.03.2357) were used for the data analysis. The NIST17 Library Search was used to identify the analyzed urinary volatile compounds that were present in the urine samples. This library search software identified each peak with the peak area and overall matching quality (%). Matching quality (%) reflects the spectral similarity between the unknown and library compounds. To minimize the risk of misidentification, only matches with a quality ≥ 50% were considered [41,50].

### 2.6. Statistical Data Analysis

All statistical analyses were performed using MetaboAnalyst 6.0, an R-based online open-source software for comprehensive metabolomics data analysis [51]. Partial Least Squares Discriminant Analysis (PLS-DA) was employed to analyze the urinary volatile organic compounds (VOCs) profiles from prostate cancer patients, aiming to differentiate between various clinical groups. The relative quantity of each peak was log-transformed (base 10) prior to performing the PLS-DA analysis. This method enables the identification of latent variables (components) that explain the variance in the data, and aids in distinguishing between the groups under investigation (e.g., pre-RP vs. post-RP, or biochemical recurrence vs. recurrent metastasis).

Variable importance in projection (VIP) scores were used to assess the contribution of each VOC to the overall model. The VIP scores were visualized using a VIP loading plot, which highlights the most influential VOCs for classification purposes.

To screen for significant VOCs, we applied both the two-sample *t*-test and the Wilcoxon rank-sum test. The *t*-test is used when the data are normally distributed, and the Wilcoxon rank-sum test is a non-parametric alternative for comparing two independent groups when normality assumptions are not met. Both tests were used to assess the significance of each VOC between different clinical groups, with a significance level set at *p* < 0.05. For multiple comparisons, False Discovery Rate (FDR) correction was applied to control for Type I errors due to the large number of tests performed.

## 3. Results

In this study, 165 urine samples were collected from males aged 45–80 years, including 55 PCa-positive pre-RP, 55 post-RP, and 55 PCa-negative controls. The post-RP samples were further classified as recovered healthy (RCH), biochemical recurrence (BCR), or recurrent metastasis (RCM). VOC profiling, metabolomics, and machine learning were used to assess biochemical changes (Table 1).

### 3.1. VOCs Extraction and Identification

To determine and identify the corresponding VOCs that are present in the respective urine samples investigated in this study, the urinary VOC extraction and sample preparation procedures previously reported by our research group were employed [41,43,50]. The extracted VOCs were analyzed using GC-MS coupled with a thermal desorption unit in scan mode. Compound identification was based on the mass spectrometry measurements, and compound abundance was determined from the instrument response signals by calculating the area under each chromatographic peak. Mirex was used as the internal standard (IS) to compute the relative abundance of each VOC that was extracted from the urine. Mirex was selected due to its minimal in vivo interference with urinary VOCs. The relative intensity of each VOC was normalized to that of Mirex, enabling semi-quantitative analysis based on the peak area ratios. Across the 165 urine samples analyzed, a total of 11,178 unique VOCs were identified.

### 3.2. Partial Least Squares Discriminant Analysis (PLS-DA) Multivariate Model

The dataset of 11,178 VOCs identified from the 165 urine samples was subjected to the PLS-DA multivariate statistical analysis model, which was applied to investigate the relationships between many different attributes. It was explored in medical diagnostics to identify disease signatures based on VOCs or other biomolecular data, in chemometrics to analyze complex chemical compositions for classification and quality control, and in genomics to classify gene expression profiles towards understanding different disease states or biological conditions. Consequently, this model was used to (1) investigate a PCa diagnosis (biopsy-designated-positive against biopsy-designated-negative PCa), (2) discriminate between pre- and post-radical prostatectomy, and (3) differentiate between recovery healthy patients, those with biochemical recurrence, and the metastatic recurrent patients post-RP.

#### 3.2.1. Prostate Cancer Diagnosis (Biopsy-Designated-Positive Against Biopsy-Designated-Negative PCa)

In the first stage of the analysis, a PCa diagnosis was considered to distinguish between the biopsy-designated-positive and biopsy-designated-negative PCa patient samples. The fifty-five biopsy-designated-negative PCa patients’ and the fifty-five biopsy-designated-positive PCa patients’ urinary VOCs were compared by subjecting the extracted VOCs data to a multivariate PLS-DA model.

**Figure 1:**
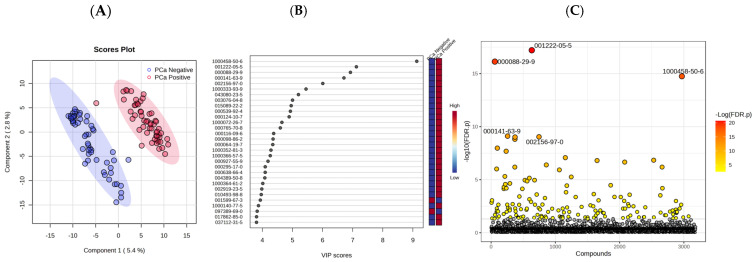
(**A**) PLS-DA score plot of biopsy-designated-positive and biopsy-designated-negative PCa. (**B**) The VIP loading plot represents the variable importance in projection (VIP) of each metabolite, while the vertical colored boxes on the right of the VIP loading plot indicate the relative concentrations of the corresponding metabolite in each group. (**C**) Fold change plot of significant VOCs in biopsy-designated-positive and biopsy-designated-negative PCa samples (*p* < 0.05). The mean concentration of each significant metabolite is represented with red- or yellow-colored dots, while the non-significant metabolites are indicated with black-colored dots. The names and chemical formula of the VOCs in the figures can be found in Supplementary Table S3.

A PLS-DA score plot was generated using the 11,178 VOCs extracted from the biopsy-designated-negative and biopsy-designated-positive PCa samples to distinguish between the two groups, as presented in Figure 1A. From the 11,178 VOCs, 155 (Supplementary Table S1) were selected by the PLS-DA algorithm as significant (*p* < 0.05). Furthermore, in order to explain the variance and distinct separation observed in the score plot, the variable importance in projection (VIP) loading plot of the top 30 most significant VOCs identified by the statistical model, with their corresponding CAS number, is illustrated in Figure 1B.

**Figure 2:**
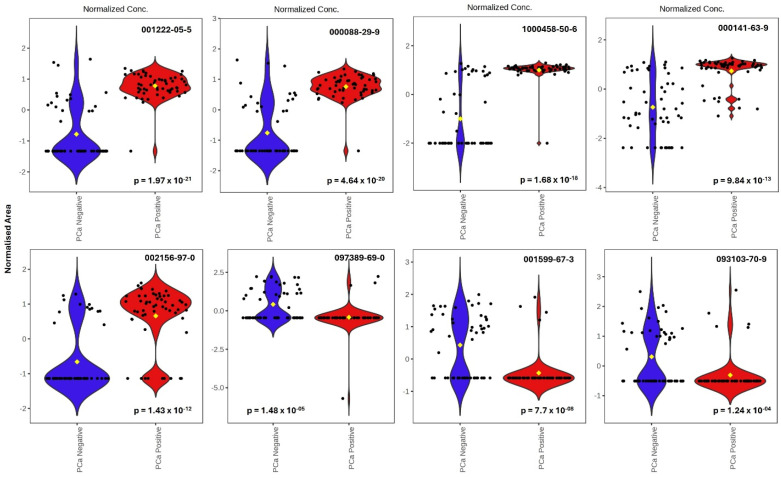
Violin plots of some significant VOCs in biopsy-designated-positive and biopsy-designated-negative PCa samples (*p* < 0.05). Blue color represents the biopsy-designated-negative PCa, while the red color indicates the biopsy-designated-positive PCa samples. CAS # 1222-05-5: Cyclopenta [g]-2-benzopyran, 1,3,4,6,7,8-hexahydro-4,6,6,7,8,8-hexamethyl-; CAS # 88-29-9: 7-Acetyl-6-ethyl-1,1,4,4-tetramethyltetralin; CAS # 1000458-50-6: 1,3,5,7,9-Pentasiloxane, 1,1,3,3,5,5,7,7,9,9-decamethyl-1,9-di (tert.butyl)-; CAS # 141-63-9: Pentasiloxane, dodecamethyl-; CAS # 2156-97-0: Dodecyl acrylate; CAS # 97389-69-0: 2′-Hydroxy-5′-methylacetophenone, TMS derivative; CAS # 1599-67-3: 1-Docosene; CAS # 93103-70-9: 2-(Acetoxymethyl)-3-(methoxycarbonyl)biphenylene.

A univariate analysis was carried out using a *t*-test, and a fold change (fc) plot was generated (Figure 1C). The univariate analysis result for each variable (i.e., VOC metabolite) was computed, and the *p*-value (*p* < 0.05) was calculated using a *t*-test with the percentage (%) of occurrence to discriminate between the biopsy-designated-negative and biopsy-designated-positive PCa samples using the relative concentrations of the corresponding metabolites in each of the groups. For a given comparison, a positive fc value indicates an increase in expression, while a negative fc indicates a decrease in expression. By examining the fc plot, the VOCs with *p*-values (and FDR *p*-values) less than a 0.05 significance threshold are significant, while the VOCs with *p*-values (and FDR *p*-values) above the significance threshold are not. The fc plot in Figure 1C shows the significant VOCs that were selected and identified by the model (the red and orange dots in the plot). In addition, violin plots of some of the top significant VOCs in discriminating PCa biopsy-designated-negative and -positive urine samples are shown in Figure 2 to demonstrate the differences in the relative concentrations of some significant VOCs when compared between the two groups.

#### 3.2.2. Distinguishing Between Pre- and Post-Radical Prostatectomy (RP)

**Figure 3:**
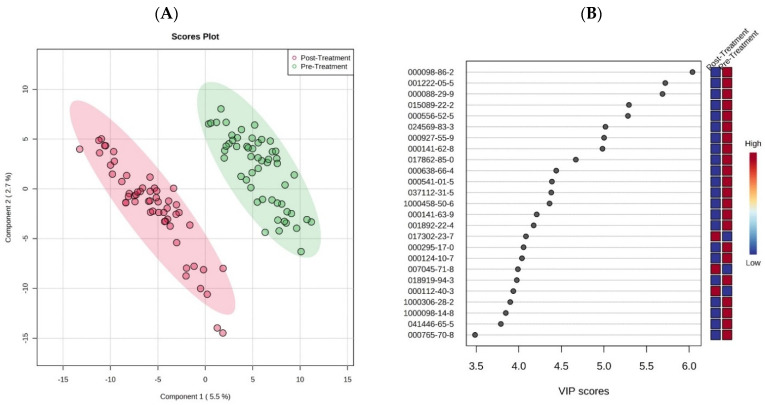
(**A**): PLS-DA score plot of pre- and post-radical prostatectomy. (**B**) The variable importance in projection (VIP) loading plot of the top 25 most significant VOCs (indicated with their corresponding CAS numbers). The colored boxes on the right of the VIP loading plot indicate the relative concentrations of the corresponding metabolites in each group. The names and chemical formula of the VOCs in the figures can be found in Supplementary Table S3.

Correspondingly, the PLS-DA multivariate analysis procedure was explored to investigate the difference in urinary VOC profiles between the patients of pre- and post-radical prostatectomy. A total of 7924 VOCs were selected by the algorithm when the pre-RP (*n* = 55) and post-RP (*n* = 55) samples were analyzed and were subjected to PLS-DA analysis. A PLS-DA score plot was generated from the data to discriminate between the two groups, as shown in Figure 3A, and the variable importance in projection (VIP) loading plot of the top 25 most significant VOCs (indicated with their corresponding CAS numbers) from the total 157 VOCs (Supplementary Table S2) is listed in Figure 3B. The colored boxes on the right of the VIP loading plot indicate the relative concentrations of the corresponding metabolites in each group. From this result, it could be inferred that the significant VOCs were able to distinguish between the pre- and post-surgery status of the patients, and also considering the clear separation observed in Figure 3A. Thus, the urinary VOCs analyzed on GC-MS could be used to monitor pathophysiological changes in the urine of PCa patients pre- and after post-radical prostatectomy.

#### 3.2.3. Distinguishing the Different Post-Radical Prostatectomy (RP) Outcomes

To examine the pathophysiological changes that could have occurred after the PCa surgery, the post-RP samples were investigated and compared with the pre-RP samples. After the surgery, it was observed that some of the patients were tested and confirmed to be PCa-free (labeled as recovered healthy—RCH, *n* = 43), while a few presented with a biochemical recurrence (BCR, *n* = 4), and some of the patients had a recurrent metastasis (RCM, *n* = 8). A total of 3984 VOCs were identified in the post-treatment cohort and were subjected to the analysis.

**Figure 4:**
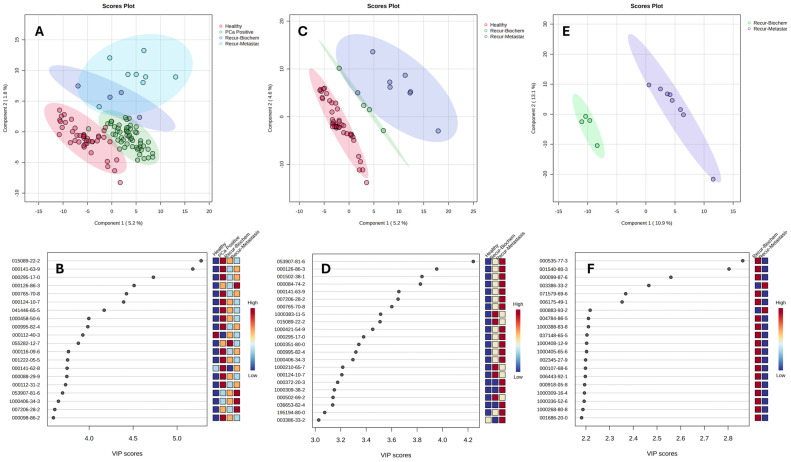
(**A**) PLS-DA and (**B**) VIP score plots of the biopsy-designated-positive PCa (labeled PCa positive, before surgery), compared with the post-radical prostatectomy recovered healthy (labeled healthy), biochemical recurrence (labeled Recur-Biochem), and recurrent metastasis of the PCa (labeled Recur-Metastasis) patients’ samples. (**C**) PLS-DA and (**D**) VIP score plots of recovered healthy, biochemical recurrence, and recurrent metastasis post-radical prostatectomy. (**E**) PLS-DA and (**F**) VIP score plots of comparison between biochemical recurrence and recurrent metastasis post-radical prostatectomy. The names and chemical formula of the VOCs in the figures can be found in Supplementary Table S3.

Figure 4A shows the PLS-DA and VIP score plots of the urinary VOCs of biopsy-designated-positive PCa (before surgery) compared with those of post-RP recovered healthy, BCR, and RCM patients. The VIP score plot presented the top 20 most significant VOCs observed to have an influence in distinguishing the differences seen in Figure 4B. In addition, it can be observed from Figure 4A that the RCH patients (red-colored dots) were well separated from the pre-RP (green dots), BCR (purpled-colored dots), and RCM groups (light blue-colored dots).

To further examine the post-radical prostatectomy groups without including the pre-RP ones, the RCH, BCR, and RCM groups were compared. Figure 4C represents the PLS-DA score plot, while Figure 4D shows the VIP score plot of 22 VOCs that are significantly different among the RCH (which are labeled healthy in the figure), BCR, and RCM. In Figure 4C, it can be observed that the RCH group was clearly separated from the biochemical recurrence and recurrent metastasis groups. This indicates that some VOCs were either depleted or overexpressed in the metastasis groups and vice versa for the recovered healthy group when compared.

Similarly, the BCR and RCM post-RP groups were compared to examine the potential difference between the two groups. Figure 4E,F shows the PLS-DA and VIP score plots of the comparison specifically between these two groups. It can be seen that the urinary VOCs were able to discriminate between the two metastasis groups successfully, as observed in Figure 4E. Table 2 provides details of the 25 significant VOCs (*p* < 0.05). Moreover, these 25 VOCs played key roles in differentiating between these two groups of PCa recurrence. Thus, these significant VOCs could be explored in developing a diagnostic tool for monitoring PCa patients after a radical prostatectomy to determine and identify patients with recurrent metastasis, which will invariably inform the physician in time on the next form of interventions that the patient will require. The results in the PLS-DA models highlight that the VOCs could result from disease-related biological pathways.

**Figure 5:**
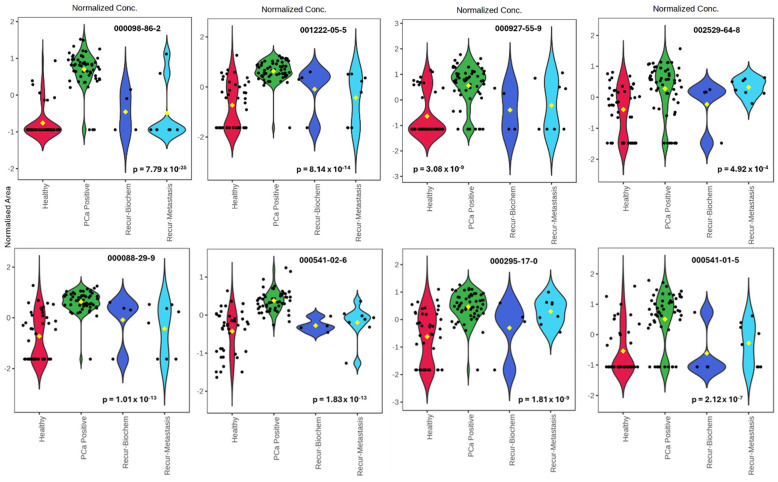
Violin plots of some significant VOCs in healthy (red color), biopsy-designated-positive (green-colored), biochemical recurrence (blue color), and recurrent metastasis (light blue colored) samples (*p* < 0.05) post-radical prostatectomy. CAS# 000098-86-2: Acetophenone; CAS# 1222-05-5: Cyclopenta [g]-2-benzopyran, 1,3,4,6,7,8-hexahydro-4,6,6,7,8,8-hexamethyl-; CAS# 927-55-9: 1-Pentanol, 4-amino-; CAS# 2529-64-8: 17beta-Estradiol, 3-deoxy-; CAS# 88-29-9: 7-Acetyl-6-ethyl-1,1,4,4-tetramethyltetralin; CAS# 541-02-6: Decamethylcyclopentasiloxane; CAS# 295-17-0: Cyclotetradecane; CAS# 541-01-5: Heptasiloxane, hexadecamethyl.

Some significant VOCs in the post-radical prostatectomy recovered healthy (labeled healthy and are red colored), biopsy-designated positive before treatment (labeled PCa positive and are green colored), biochemical recurrence post-treatment (labeled Recur-Biochem and are blue colored), and recurrent metastasis post-treatment (labeled Recur-Metastasis and are light blue colored) samples (*p* < 0.05) post-radical prostatectomy are further illustrated in Figure 5’s violin plots. The plots show the variations in some representative VOC metabolites that were found in the respective groups.

## 4. Discussion

PLS-DA is a powerful multivariate technique that combines aspects of regression and classification, allowing for dimensionality reduction while maximizing the variance between groups. It is particularly effective in handling high-dimensional data, like VOCs, where the number of features (variables) often exceeds the number of observations. This method enables the identification of latent variables (components) that explain the variance in the data and aid in distinguishing between the groups under investigation (e.g., pre-treatment vs. post-treatment, or biochemical recurrence vs. recurrent metastasis). In this study, the PLS-DA model enables effective classification and visualization while maintaining data integrity. The model further extracts components that explain the variance in the predictor variables, which are most useful for differentiating between the classes. The variable importance in projection (VIP) scores are an essential characteristic of PLS-DA when it comes to feature selection. VIP scores help identify influential variables (e.g., VOCs) in distinguishing between classes or outcomes in the model. The VIP scores are calculated by weighing the contribution of each variable to the model’s ability to explain the response variable Y. To interpret the scores, a VIP score close to or greater than 1 indicates that the variable is considered important in the projection used in the PLS-DA model. Thus, the VIP loading plots (such as Figure 1B and Figure 3B) denote the relative contribution of the VOCs to the variance between the PCa biopsy-designated-negative and biopsy-designated-positive urine samples and pre- and post-treatment samples.

### 4.1. Application of Urine VOCs Selected by PLS-DA Models in Class Differentiation

In this study, urine samples from three different groups were obtained as follows: (1) 55 samples from patients with biopsy-designated PCa-positive results before a radical prostatectomy (RP), (2) 55 samples from the same patient cohort after a RP, and (3) 55 urine samples from patients with biopsy-designated PCa-negative (control). As illustrated in Figure 1A and Figure 3A, the urinary VOC profiles effectively differentiate between PCa–negative and PCa–positive patients, as well as between pre- and post-treatment groups. Moreover, 13 of the top 30 VOCs that most significantly differentiated between PCa-positive and PCa-negative groups (Figure 1B) were also identified as significant in the comparison between pre- and post-RP cohorts (Figure 3B), suggesting that the urinary VOC profiles of post-treatment patients resemble those of PCa-negative individuals, potentially reflecting the biochemical normalization associated with tumor removal.

The outcomes of patients who underwent a RP varied. The 55 post-RP patients were classified into three groups based on their post-surgical health status as follows: recovered healthy (RCH), biochemical recurrence (BCR), and recurrent metastasis (RCM). Figure 4C,E further demonstrates distinct clustering among the three groups as well as between the BCR and RCM groups. As shown in Figure 4D, the 22 most significant VOCs were reduced in the RCH group compared with the BCR and RCM groups, with their identities listed in Supplementary Table S3. Despite the small sample sizes in the BCR (*n* = 4) and RCM (*n* = 8) groups, the PLS-DA model identified 25 VOCs (Table 2) that significantly differed between them. The compounds identified in the figures and tables may reflect complex metabolic alterations that are associated with prostate and other cancers. These findings support the potential of urinary VOCs for non-invasive detection of PCa.

### 4.2. Biological Significance of Selected VOCs

Numerous urinary VOCs showed statistically significant differences in abundance between PCa-positive and PCa-negative patients, as well as between pre- and post-treatment groups. Although the origins of these VOCs remain unclear and are beyond the scope of this exploratory study, we further investigated the biological significance of several compounds, particularly those that distinguish pre- from post-treatment groups and those associated with treatment outcomes such as BCR and RCM.

To better understand these findings, some of the significant VOCs highlighted in Figure 1B, Figure 2, Figure 3B and Figure 4C,E, and Table 2 were categorized by their chemical class, each offering insight into prostate cancer progression. For example, the ketone compounds found in Figure 1B (e.g., 2-Propanone, 1-hydroxy-(CAS 116-09-6), and Acetophenone (CAS 98-86-2), 2′-Hydroxy-5′-methylacetophenone, TMS derivative (CAS 97389-69-0 in Figure 2) and in Table 2 (e.g., 2-dodecanone, 2-tetradecanone) are likely linked to fatty acid β-oxidation and mitochondrial adaptation under hypoxia, which is consistent with the Warburg Effect [52]. Aldehydes (e.g., Octadecanal (CAS 638-66-4; Figure 1B), Octadecanal (CAS 638-66-4 in Figure 3B, and Benzaldehyde in Table 2), and hydrocarbons (e.g., Cyclotetradecane (CAS 295-17-0 in Figure 5, and cymenes in Table 2)) are indicative of lipid peroxidation, which is caused by elevated reactive oxygen species (ROS) in cancer [53]. Esters (such as Fumaric acid, 2-methylpentyl tridec-2-yn-1-yl ester in Table 2) suggest lipid remodeling, detoxification, or microbial metabolism, as well as TCA cycle disruption and onco-metabolic signaling, which are particularly linked to Fumarate Hydratase (FH) deficiency and HIF-1α stabilization [54,55,56]. Nitrogen- and sulfur-containing molecules (e.g., DOMA, diethylenetriamine, N,N-Dimethylmethane solfonamide) are associated with amino acid catabolism, catecholamine turnover, and immune-metabolic stress [57,58]. Similarly, the detection of various VOCs, such as Siloxanes and inorganic species like silicotungstic acid, identified in the urine (Table 2), points to the upregulation of cytochrome P450-mediated detoxification pathways in cancer, which impact the specific VOC signature observed in these patients [59,60]. Together, this chemical and biological categorization underscores the value of VOCs as functional readouts of cancer metabolism, immune signaling, microbiome crosstalk, and environmental interactions. The patterns observed here reinforce the promise of VOC profiling as a non-invasive tool for distinguishing prostate cancer recurrence phenotypes. Some metabolic pathways are further discussed as follows.

#### 4.2.1. Hydrocarbons and Aldehydes Metabolism

Hydrocarbon compounds, such as Cyclotetradecane (CAS 295-17-0, in Figure 1B), 1-Docosene (CAS 1599-67-3 in Figure 1B), Undecane, 2-methyl-(CAS 7045-71-8 in Figure 3B), Nonane, 4,5-dimethyl- (CAS 17302-23-7, in Figure 3B), meta cymene, para cymene, octadecane, and the chlorinated or unsaturated tetradecene derivatives in Table 2 were found significant when comparing the urine VOCs profiles between the PCa positive and negative groups, pre- and post-treatment, and even among different treatment outcomes. Hydrocarbons and aldehydes are consistent with reactive oxygen species (ROS)-induced membrane lipid degradation, reflecting the elevated ROS levels that are commonly observed in cancer cells. Excessive ROS generation contributes to lipid peroxidation, producing alkanes and aldehydes as byproducts of polyunsaturated fatty acid degradation in cellular membranes [61,62]. Cancer cells often maintain a delicate balance of ROS, while low-to-moderate levels of ROS promote proliferation and genomic instability, and excessive ROS induces oxidative damage, which can sensitize cells to treatment or trigger cell death [63,64]. Thus, the detection of volatile hydrocarbons and aldehydes in urine or breath may reflect ongoing oxidative stress and tumor-associated redox imbalance.

Benzaldehyde was found to be significant in differentiating the BCR and RCM groups (Table 2). As such, it is a simple aromatic aldehyde, and it has been shown in vitro to suppress multiple oncogenic signaling pathways, including PI3K/Akt/mTOR, NF-κB, STAT3, and ERK, by disrupting the 14-3-3-mediated protein interactions in pancreatic (BxPC-3) and non-small-cell lung cancer (A549) cell lines. This inhibition impairs epithelial–mesenchymal plasticity (EMP) and has been associated with reduced therapy resistance [65].

#### 4.2.2. Ketones, Esters, and Alcohols Metabolism

In Figure 1B and Figure 3B, ketones such as Acetophenone (CAS 98-86-2), and 2-Propanone, 1-hydroxy-(CAS 116-09-6) were found at higher levels in the urine of PCa-positive or pre-treatment groups, and most probably arose from the enhanced fatty acid β-oxidation or ω-oxidation processes, reflecting the metabolic adaptations that were employed by cancer cells under hypoxic stress to maintain energy production. These VOCs serve as indirect markers of mitochondrial reprogramming and lipid catabolism in tumor microenvironments [66,67]. 2-Dodecanone and 2-tetradecanone (in Table 2) have been identified as products of intensified enhanced fatty acid β-oxidation activity—a hallmark of tumor metabolic plasticity [68].

Acetophenone consistently emerged as a significant VOC in comparisons between PCa-positive and PCa-negative patients, between pre- and post-treatment groups, and among post-treatment subgroups (Supplementary Table S3). Previous studies have also highlighted its relevance as a biomarker: Acetophenone in exhaled breath has been reported to be an important biomarker of breast cancer [69]. In our previous study [41], acetophenone in urine was also found to be significantly different between PCa-positive and PCa-negative patients. Acetophenone in saliva was identified as a significant biomarker for Hepatocellular carcinoma [70]. It could be due to oxidative stress, which is a common denominator in the pathogenesis of cancer and other chronic diseases [41].

Short-chain esters and alcohols, meanwhile, may reflect lipid metabolism remodeling or detoxification conjugation. These compounds, therefore, suggest shifts toward alternative energy sources in malignancies where glycolytic or mitochondrial metabolism is compromised. Additionally, esterified VOCs may result from phase II conjugation detoxification, lipid esterification, or microbial degradation, highlighting the complexity of biochemical sources and interactions that give rise to these volatile biomarkers [71,72]. In Figure 4B,D, where VOC profiles in patients with different post-treatment outcomes were compared, several esters and alcohols, such as Methyl tetradecanoate, Carbonic acid, octadecyl prop-1-en-2-yl ester, 1-Hexadecanol, 3-Fluorophenol, and Oxalic acid, isobutyl heptadecyl ester (Supplementary Table S3), were found to be at the lowest levels in recovered healthy patients as compared to the BCR and RCM groups.

#### 4.2.3. Nitrogen- and Sulfur-Containing Molecules in Cancer Metabolism

Among the 25 VOCs that significantly differentiated BCR from RCM (Table 2), several sulfur- and nitrogen-containing compounds—including 2-(methylamino)ethane sulfonic acid, sulfurous acid, pentadecyl 2-pentyl ester, and N,N-dimethylmethane sulfonamide—were detected at significantly higher levels in the urine of the BCR group compared with the RCM group. Nitrogen and sulfur are essential bioelements that contribute significantly to the biochemical architecture of cellular metabolism, particularly in the context of cancer-associated physiological reprogramming. In tumors, the altered metabolic landscape reflects increased demands for nitrogen- and sulfur-containing substrates to support cellular proliferation, redox balance, epigenetic remodeling, and immune evasion [73,74]. As cancer progresses, these demands result in the restructuring of amino acid catabolism, modification of neurotransmitter pathways, and activation of stress- and immune-related signaling mechanisms, often leading to the accumulation of distinct nitrogen- and sulfur-containing small molecules that are detectable in bodily fluids such as urine and plasma [57,58,74]. Among these compounds, 2-(methylamino)-ethane sulfonic acid, N,N-dimethylmethane sulfonamide, diethylenetriamine, and 3,4-dihydroxymandelic acid (DOMA) are particularly prominent. These metabolites may originate from amino acid degradation, sulfonic acid metabolism, and polyamine turnover, all of which are known to be dysregulated in the tumor microenvironment [75]. For instance, DOMA, a major urinary metabolite of noradrenaline, reflects sympathetic nervous system activation, catecholamine catabolism, and oxidative stress adaptation, all hallmarks of the neuroimmune crosstalk and metabolic stress that are characteristic of aggressive cancers [57,58]. DOMA also exhibits strong antioxidative potential, potentially contributing to the redox buffering systems in tumors that are exposed to elevated oxidative burden [75].

Moreover, polyamine analogs, such as diethylenetriamine and sulfonamide derivatives, which were present at higher levels in the BCR group compared with the RCM group (Table 2), may reflect enhanced cellular detoxification processes, nucleic acid biosynthesis, and stress-induced methylation cycles—mechanisms that collectively contribute to tumor growth, adaptation, and resistance [76,77]. These molecules often serve dual roles as metabolic intermediates and signaling molecules, modulating both immune cell recruitment and cancer cell survival. Hence, profiling and detection of nitrogen- and sulfur-rich VOCs in urine may provide a non-invasive window into the complex metabolic rewiring of malignancies, shedding light on the interconnected pathways of inflammation, neurotransmitter turnover, immune adaptation, and tumor stress response [74,78]. This work aimed to highlight the potential application of urinary VOCs in monitoring PCa post-radical prostatectomy. The rationale lies in the hypothesis of urinary VOCs being the reflection of physiological status in humans and thus could serve as biomarkers for detecting and monitoring prostate cancer recurrence post a radical prostatectomy, towards achieving the following:

1. Early Detection: VOCs are small molecules that can be released into urine through metabolic processes or other biological pathways that are associated with cancer cells. Changes in VOC profiles may occur early in the progression of disease, potentially allowing for earlier detection of recurrence compared to traditional methods.
2. Non-invasive Monitoring: A radical prostatectomy is a common treatment for localized prostate cancer. After surgery, the primary concern is monitoring for cancer recurrence. Current monitoring methods, such as PSA testing and imaging techniques, have limitations. The use of VOCs in urine offers a non-invasive approach that could complement or improve existing methods.
3. Mass Spectrometry Precision: Mass spectrometry is a highly sensitive and specific analytical technique capable of detecting and quantifying VOCs in biological samples such as urine. This technology allows for the identification of specific VOC profiles that correlate with prostate cancer status, providing a reliable method for monitoring patients post a radical prostatectomy.
4. Personalized Medicine: The identification of distinct VOC signatures associated with prostate cancer recurrence can facilitate personalized treatment strategies. By monitoring VOC profiles over time, clinicians may tailor interventions more effectively, including the timing of adjuvant therapies or interventions aimed at preventing disease progression.
5. Research and Clinical Translation: Previous studies have shown promising results regarding the feasibility and accuracy of using VOC analysis for cancer monitoring. Further research aims to validate these findings in larger cohorts, establish standardized protocols, and potentially integrate VOC analysis into routine clinical practice as a complementary diagnostic tool.

The limitations of this study include the following: (1) The number of samples was small. Thus, there was no independent validation that could be conducted. (2) Many VOCs were not detected in every subject. For handling missing data, we initially imputed missing VOC values with zeroes. However, future studies with a larger cohort size may consider alternative imputation methods, such as a mean imputation or k-nearest neighbor imputation, which could provide a more robust solution by considering the relationships between observed values and reducing bias. (3) For the VOC markers with significant differences between the comparison groups, this study only performed PLS-DA analysis. (4) This study is also limited by the absence of specific normalization to account for urine dilution. While analyses were performed using relative abundances and uniform sample handling protocols, the potential influence of inter-individual variations in water intake and exogenous factors on metabolite levels cannot be entirely ruled out.

This study demonstrates the potential of urinary VOC profiling combined with metabolomics and machine learning to differentiate prostate cancer patients before and after a radical prostatectomy and to explore the recurrence status. While the results highlight metabolites and patterns of interest, the small subgroup sizes and the absence of an independent validation cohort limit the statistical strength and translational impact of the findings. Therefore, these results should be considered preliminary and hypothesis-generating, providing a foundation for future studies with larger cohorts and external validation to confirm and extend these observations.

## 5. Conclusions

The application of urinary VOCs profiling has garnered attention as a potential non-invasive method for disease monitoring in various medical conditions, including prostate cancer. The use of mass spectrometry analysis of urinary VOCs for monitoring prostate cancer post a radical prostatectomy fills the clinical needs to identify biomarkers for more sensitive, specific, and non-invasive methods to detect cancer recurrence early and, thus, improve patient outcomes. After a radical prostatectomy, traditional methods often involve blood tests (PSA levels) or imaging scans. However, these methods may not always be sensitive or accessible to detect early signs of recurrence. Thus, urinary VOC profiling holds potential as a non-invasive method for disease monitoring in patients with prostate cancer after a radical prostatectomy.

Further research and development are necessary to refine the techniques, establish standards, and validate their clinical utility in this context. Standardization of sample collection and analysis methods is essential to ensure reproducibility and reliability of results. Additionally, larger-scale clinical studies are needed to validate the accuracy and sensitivity of VOC profiling compared to existing diagnostic monitoring methods.

## Figures and Tables

**Table 1:** Age and racial distributions of patients of biopsy-designated positive PCa pre- and post-radical prostatectomy and biopsy-designated negative PCa (healthy control) subjects.

		Control	Pre-Treatment	Black Americans (Post-Treatment)			White (Post-Treatment)		
Age Bracket (Years)	Total	PCa Negative	PCa Positive	RCH	BCR	RCM	RCH	BCR	RCM
45–50	14	8	6	1	**	1	4	**	**
51–55	25	16	9	1	**	1	6	**	1
56–60	23	12	11	3	**	**	7	1	**
61–65	25	7	18	2	1	3	10	**	2
66–70	14	4	10	3	**	**	5	2	**
71–75	6	5	1	**	**	**	1	**	**
76–80	3	3	0	**	**	**	**	**	**
Total	110	55	55	10	1	5	33	3	3

** Denotes zero value; RCH: recovered healthy; BCR: biochemical recurrence; RCM: recurrent metastasis.

**Table 2:** Significant VOCs identified when biochemical recurrence (BCR) and recurrent metastasis (RCM) were compared (*p* < 0.05, obtained from the Wilcoxon test).

BCR vs. RCM	CAS Number	Compound Name	*p*-Value	Higher in
1	000535-77-3	*meta*-Cymene	1.13 × 10^−3^	BCR
2	001540-80-3	1,8-Cyclotetradecadiyne	3.02 × 10^−2^	BCR
3	000099-87-6	*para*-Cymene	6.80 × 10^−3^	BCR
4	003386-33-2	1-chloro-Octadecane	1.04 × 10^−2^	RCM
5	071579-69-6	Tetrasiloxane	1.55 × 10^−2^	BCR
6	006175-49-1	2-dodecanone	1.65 × 10^−2^	BCR
7	000883-93-2	Benzaldehyde	2.67 × 10^−2^	RCM
8	004784-86-5	1,2-dimethylcyclopentadiene	3.25 × 10^−2^	BCR
9	1000388-83-8	5-Methoxy-2-methyl-9-oxa-1-azatetracyclo [8.7.0.0(3,8).0(11,16)]heptadeca3(8),4,6,11(16),12,14-hexaen-17-one	2.74 × 10^−2^	BCR
10	037148-65-5	3,4-dihydroxylmandelic acid	4.50 × 10^−2^	BCR
11	1000408-12-9	2-{[(Trimethylsilyl)oxy]carbonyl}phenyl 2-[(trimethylsilyl)oxy]benzoate	2.80 × 10^−2^	BCR
12	1000405-65-6	Fumaric acid, 2-methylpentyl tridec-2-yn1-yl ester	2.81 × 10^−2^	BCR
13	002345-27-9	2-Tetradecanone	2.84 × 10^−2^	BCR
14	000107-68-6	2-(Methylamino)ethane sulfonic acid	2.85 × 10^−2^	BCR
15	006443-92-1	Cis-2-heptene	2.86 × 10^−2^	BCR
16	000918-05-8	N,N-Dimethylmethane solfonamide	4.54 × 10^−2^	BCR
17	1000309-16-4	Sulfurous acid, pentadecyl 2-pentyl ester	2.89 × 10^−2^	BCR
18	1000336-52-6	Octadecane-1,2-diol, 2TMS derivative	2.90 × 10^−2^	BCR
19	1000268-80-8	Pyrazol-5(4H)-one, 1-acetyl-4-allyl-3-methyl	2.95 × 10^−2^	BCR
20	001686-20-0	*para*-Mentha-1,5-dien-8-ol	3.01 × 10^−2^	BCR
21	000126-86-3	2,4,7,9-Tetramethyl-5-decyne-4,7-diol	3.36 × 10^−2^	RCM
22	002425-54-9	1-Chlorotetradecane	3.40 × 10^−2^	BCR
23	000112-05-0	Pelargonic acid	3.81 × 10^−2^	RCM
24	000111-40-0	Di-ethylenetriamine	4.76 × 10^−2^	BCR
25	020634-43-9	Silicotungstic acid	4.85 × 10^−2^	BCR

## Data Availability

The raw data supporting the conclusions of this article will be made available by the authors on request.

## Supplementary Materials

The following supporting information can be downloaded at: https://www.mdpi.com/article/10.3390/cancers17172756/s1, Supplementary Table S1: 155 VOCs that demonstrate significant differences (*p* < 0.05) between the biopsy-designated-positive and biopsy-designated-negative PCa groups generated by PLS-DA; Supplementary Table S2: 157 VOCs that demonstrate significant differences (*p* < 0.05) between the pre- and post-RP groups generated by PLS-DA; Supplementary Table S3: The Compounds Names of the VOCs included in Figure 1, Figure 2, Figure 3, Figure 4 and Figure 5.

## Abbreviations

The following abbreviations are used in this manuscript:

AUC	Area Under Curve
BCR	Biochemical Recurrence
CIS	Cryogenic Injection System
DI	Deionized
fc	Fold Change
FDR	False Discovery Rate
GC-MS	Gas Chromatography-Mass Spectrometry
IS	Internal Standard
RCM	Recurrent Metastasis
NIST	National Institute Of Standards And Technology
PCa	Prostate Cancer
PLS-DA	Partial Least Squares Discriminant Analysis
PSA	Prostate-Specific Antigen
RCH	Recovered Healthy
ROC	Receiver Operating Characteristic Curve
RP	Radical Prostatectomy
SBSE	Stir Bar Sorptive Extraction
TDT	Thermal Desorption Tube
TDU	Thermal Desorption Unit
VIP	Variable Importance In Projection
VOCs	Volatile Organic Compounds

## References

[1] Siegel R.L., Kratzer T.B., Giaquinto A.N., Sung H., Jemal A. (2025). Cancer Statistics, 2025. CA Cancer J. Clin..

[2] Bekelman J.E., Rumble R.B., Chen R.C., Pisansky T.M., Finelli A., Feifer A., Nguyen P.L., Loblaw D.A., Tagawa S.T., Gillessen S. (2018). Clinically Localized Prostate Cancer: ASCO Clinical Practice Guideline Endorsement of an American Urological Association/American Society for Radiation Oncology/Society of Urologic Oncology Guideline. J. Clin. Oncol..

[3] Doan P., Scheltema M.J., Amin A., Shnier R., Geboers B., Gondoputro W., Moses D., Van Leeuwen P.J., Haynes A.M., Matthews J. (2022). Final Analysis of the Magnetic Resonance Imaging in Active Surveillance Trial. J. Urol..

[4] Eklund M., Jäderling F., Discacciati A., Bergman M., Annerstedt M., Aly M., Glaessgen A., Carlsson S., Grönberg H., Nordström T. (2021). MRI-Targeted or Standard Biopsy in Prostate Cancer Screening. N. Engl. J. Med..

[5] Evans A.J. (2018). Treatment Effects in Prostate Cancer. Mod. Pathol..

[6] Khan A., Khan A.U., Siref L., Feloney M. (2023). Focal Cryoablation of the Prostate: Primary Treatment in 163 Patients With Localized Prostate Cancer. Cureus.

[7] Nyk Ł., Michalak W., Szempliński S., Woźniak R., Zagożdżon B., Krajewski W., Kryst P., Kamecki H., Poletajew S. (2022). High-Intensity Focused-Ultrasound Focal Therapy Versus Laparoscopic Radical Prostatectomy: A Comparison of Oncological and Functional Outcomes in Low- and Intermediate-Risk Prostate Cancer Patients. J. Pers. Med..

[8] Wang I., Song L., Wang B.Y., Rezazadeh Kalebasty A., Uchio E., Zi X. (2022). Prostate Cancer Immunotherapy: A Review of Recent Advancements with Novel Treatment Methods and Efficacy. Am. J. Clin. Exp. Urol..

[9] Bach C., Pisipati S., Daneshwar D., Wright M., Rowe E., Gillatt D., Persad R., Koupparis A. (2014). The Status of Surgery in the Management of High-Risk Prostate Cancer. Nat. Rev. Urol..

[10] Kan C.K., Qureshi M.M., Gupta A., Agarwal A., Gignac G.A., Bloch B.N., Thoreson N., Hirsch A.E. (2018). Risk Factors Involved in Treatment Delays and Differences in Treatment Type for Patients with Prostate Cancer by Risk Category in an Academic Safety Net Hospital. Adv. Radiat. Oncol..

[11] Sekhoacha M., Riet K., Motloung P., Gumenku L., Adegoke A., Mashele S. (2022). Prostate Cancer Review: Genetics, Diagnosis, Treatment Options, and Alternative Approaches. Molecules.

[12] Kasivisvanathan V., Rannikko A.S., Borghi M., Panebianco V., Mynderse L.A., Vaarala M.H., Briganti A., Budäus L., Hellawell G., Hindley R.G. (2018). MRI-Targeted or Standard Biopsy for Prostate-Cancer Diagnosis. N. Engl. J. Med..

[13] Nordström T., Discacciati A., Bergman M., Clements M., Aly M., Annerstedt M., Glaessgen A., Carlsson S., Jäderling F., Eklund M. (2021). Prostate Cancer Screening Using a Combination of Risk-Prediction, MRI, and Targeted Prostate Biopsies (STHLM3-MRI): A Prospective, Population-Based, Randomised, Open-Label, Non-Inferiority Trial. Lancet Oncol..

[14] Miller K.D., Nogueira L., Devasia T., Mariotto A.B., Yabroff K.R., Jemal A., Kramer J., Siegel R.L. (2022). Cancer Treatment and Survivorship Statistics, 2022. CA. Cancer J. Clin..

[15] Siegel R.L., Miller K.D., Fuchs H.E., Jemal A. (2021). Cancer Statistics, 2021. CA. Cancer J. Clin..

[16] Wu S., Xie L., Lin S.X., Wirth G.J., Lu M., Zhang Y., Blute M.L., Dahl D.M., Wu C.-L. (2020). Quantification of Perineural Invasion Focus after Radical Prostatectomy Could Improve Predictive Power of Recurrence. Hum. Pathol..

[17] Zattoni F., Heidegger I., Kasivisvanathan V., Kretschmer A., Marra G., Magli A., Preisser F., Tilki D., Tsaur I., Valerio M. (2021). Radiation Therapy After Radical Prostatectomy: What Has Changed Over Time?. Front. Surg..

[18] Marino F., Moretto S., Rossi F., Bizzarri F.P., Gandi C., Filomena G.B., Gavi F., Russo P., Campetella M., Totaro A. (2024). Robot-Assisted Radical Prostatectomy with the Hugo RAS and Da Vinci Surgical Robotic Systems: A Systematic Review and Meta-Analysis of Comparative Studies. Eur. Urol. Focus..

[19] Bommelaere T., Villers A., Puech P., Ploussard G., Labreuche J., Drumez E., Leroy X., Olivier J. (2022). Risk Estimation of Metastatic Recurrence After Prostatectomy: A Model Using Preoperative Magnetic Resonance Imaging and Targeted Biopsy. Eur. Urol. Open Sci..

[20] Shore N.D., Moul J.W., Pienta K.J., Czernin J., King M.T., Freedland S.J. (2024). Biochemical Recurrence in Patients with Prostate Cancer after Primary Definitive Therapy: Treatment Based on Risk Stratification. Prostate Cancer Prostatic Dis..

[21] Kim W.T., Kim J., Kim W.-J. (2022). How Can We Best Manage Biochemical Failure after Radical Prostatectomy?. Investig. Clin. Urol..

[22] Albisinni S., Diamand R. (2023). Understanding Biochemical Recurrence after Radical Prostatectomy: Trust Biology, Not a Number. Prostate Cancer Prostatic Dis..

[23] Rebello R.J., Oing C., Knudsen K.E., Loeb S., Johnson D.C., Reiter R.E., Gillessen S., Van Der Kwast T., Bristow R.G. (2021). Prostate Cancer. Nat. Rev. Dis. Primer.

[24] Tourinho-Barbosa R., Srougi V., Nunes-Silva I., Baghdadi M., Rembeyo G., Eiffel S.S., Barret E., Rozet F., Galiano M., Cathelineau X. (2018). Biochemical Recurrence after Radical Prostatectomy: What Does It Mean?. Int. Braz. J. Urol..

[25] Jiang Q., Xie M., He M., Yan F., Chen M., Xu S., Zhang X., Shen P. (2019). PITX2 Methylation: A Novel and Effective Biomarker for Monitoring Biochemical Recurrence Risk of Prostate Cancer. Medicine.

[26] Parker C., Castro E., Fizazi K., Heidenreich A., Ost P., Procopio G., Tombal B., Gillessen S. (2020). Prostate Cancer: ESMO Clinical Practice Guidelines for Diagnosis, Treatment and Follow-Up. Ann. Oncol..

[27] Cheung P.K., Ma M.H., Tse H.F., Yeung K.F., Tsang H.F., Chu M.K.M., Kan C.M., Cho W.C.S., Ng L.B.W., Chan L.W.C. (2019). The Applications of Metabolomics in the Molecular Diagnostics of Cancer. Expert Rev. Mol. Diagn..

[28] Schmidt D.R., Patel R., Kirsch D.G., Lewis C.A., Vander Heiden M.G., Locasale J.W. (2021). Metabolomics in Cancer Research and Emerging Applications in Clinical Oncology. CA. Cancer J. Clin..

[29] Gonzalez-Covarrubias V., Martínez-Martínez E., del Bosque-Plata L. (2022). The Potential of Metabolomics in Biomedical Applications. Metabolites.

[30] Tounta V., Liu Y., Cheyne A., Larrouy-Maumus G. (2021). Metabolomics in Infectious Diseases and Drug Discovery. Mol. Omics.

[31] Liu R., Bao Z.-X., Zhao P.-J., Li G.-H. (2021). Advances in the Study of Metabolomics and Metabolites in Some Species Interactions. Molecules.

[32] Birhanu A.G. (2023). Mass Spectrometry-Based Proteomics as an Emerging Tool in Clinical Laboratories. Clin. Proteom..

[33] Qiu S., Guo S., Yang Q., Xie Y., Tang S., Zhang A. (2023). Innovation in Identifying Metabolites from Complex Metabolome—Highlights of Recent Analytical Platforms and Protocols. Front. Chem..

[34] Danzi F., Pacchiana R., Mafficini A., Scupoli M.T., Scarpa A., Donadelli M., Fiore A. (2023). To Metabolomics and beyond: A Technological Portfolio to Investigate Cancer Metabolism. Signal Transduct. Target. Ther..

[35] Donatti A., Canto A.M., Godoi A.B., Da Rosa D.C., Lopes-Cendes I. (2020). Circulating Metabolites as Potential Biomarkers for Neurological Disorders—Metabolites in Neurological Disorders. Metabolites.

[36] Le T., Priefer R. (2023). Detection Technologies of Volatile Organic Compounds in the Breath for Cancer Diagnoses. Talanta.

[37] Gao Q., Lee W.-Y. (2019). Urinary Metabolites for Urological Cancer Detection: A Review on the Application of Volatile Organic Compounds for Cancers. Am. J. Clin. Exp. Urol..

[38] Holbrook K.L., Lee W.-Y. (2025). Volatile Organic Metabolites as Potential Biomarkers for Genitourinary Cancers: Review of the Applications and Detection Methods. Metabolites.

[39] Oxner M., Trang A., Mehta J., Forsyth C., Swanson B., Keshavarzian A., Bhushan A. (2023). The Versatility and Diagnostic Potential of VOC Profiling for Noninfectious Diseases. BME Front..

[40] Zhang J.D., Le M.N., Hill K.J., Cooper A.A., Stuetz R.M., Donald W.A. (2022). Identifying Robust and Reliable Volatile Organic Compounds in Human Sebum for Biomarker Discovery. Anal. Chim. Acta.

[41] Badmos S. (2024). Urinary Volatile Organic Compounds in Prostate Cancer Biopsy Pathologic Risk Stratification Using Logistic Regression and Multivariate Analysis Models. Am. J. Cancer Res..

[42] Gao Q., Su X., Annabi M.H., Schreiter B.R., Prince T., Ackerman A., Morgas S., Mata V., Williams H., Lee W.-Y. (2019). Application of Urinary Volatile Organic Compounds (VOCs) for the Diagnosis of Prostate Cancer. Clin. Genitourin. Cancer.

[43] Holbrook K.L., Quaye G.E., Noriega Landa E., Su X., Gao Q., Williams H., Young R., Badmos S., Habib A., Chacon A.A. (2024). Detection and Validation of Organic Metabolites in Urine for Clear Cell Renal Cell Carcinoma Diagnosis. Metabolites.

[44] Farraia M.V., Cavaleiro Rufo J., Paciência I., Mendes F., Delgado L., Moreira A. (2019). The Electronic Nose Technology in Clinical Diagnosis: A Systematic Review. Porto Biomed. J..

[45] Scheepers M.H.M.C., Al-Difaie Z., Brandts L., Peeters A., Van Grinsven B., Bouvy N.D. (2022). Diagnostic Performance of Electronic Noses in Cancer Diagnoses Using Exhaled Breath: A Systematic Review and Meta-Analysis. JAMA Netw. Open.

[46] da Costa B.R.B., De Martinis B.S. (2020). Analysis of Urinary VOCs Using Mass Spectrometric Methods to Diagnose Cancer: A Review. Clin. Mass. Spectrom..

[47] Rondanelli M., Perdoni F., Infantino V., Faliva M.A., Peroni G., Iannello G., Nichetti M., Alalwan T.A., Perna S., Cocuzza C. (2019). Volatile Organic Compounds as Biomarkers of Gastrointestinal Diseases and Nutritional Status. J. Anal. Methods Chem..

[48] Boulind C.E., Gould O., De Lacy Costello B., Allison J., White P., Ewings P., Wicaksono A.N., Curtis N.J., Pullyblank A., Jayne D. (2022). Urinary Volatile Organic Compound Testing in Fast-Track Patients with Suspected Colorectal Cancer. Cancers.

[49] Liu Q., Fan Y., Zeng S., Zhao Y., Yu L., Zhao L., Gao J., Zhang X., Zhang Y. (2023). Volatile Organic Compounds for Early Detection of Prostate Cancer from Urine. Heliyon.

[50] Noriega Landa E., Quaye G.E., Su X., Badmos S., Holbrook K.L., Polascik T.J., Adams E.S., Deivasigamani S., Gao Q., Annabi M.H. (2024). Urinary Fatty Acid Biomarkers for Prostate Cancer Detection. PLoS ONE.

[51] Ewald J.D., Zhou G., Lu Y., Kolic J., Ellis C., Johnson J.D., Macdonald P.E., Xia J. (2024). Web-Based Multi-Omics Integration Using the Analyst Software Suite. Nat. Protoc..

[52] Vander Heiden M.G., Cantley L.C., Thompson C.B. (2009). Understanding the Warburg Effect: The Metabolic Requirements of Cell Proliferation. Science.

[53] Panieri E., Santoro M.M. (2016). ROS Homeostasis and Metabolism: A Dangerous Liason in Cancer Cells. Cell Death Dis..

[54] Ashrafian H., O’Flaherty L., Adam J., Steeples V., Chung Y.-L., East P., Vanharanta S., Lehtonen H., Nye E., Hatipoglu E. (2010). Expression Profiling in Progressive Stages of Fumarate-Hydratase Deficiency: The Contribution of Metabolic Changes to Tumorigenesis. Cancer Res..

[55] Sciacovelli M., Gonçalves E., Johnson T.I., Zecchini V.R., da Costa A.S., Gaude E., Frezza C. (2016). Fumarate Is an Epigenetic Modifier That Elicits Epithelial to Mesenchymal Transition. Nature.

[56] Valcarcel-Jimenez L., Frezza C. (2023). Fumarate Hydratase (FH) and Cancer: A Paradigm of Oncometabolism. Br. J. Cancer.

[57] Nagatsu T. (2006). The catecholamine system in health and disease—Relation to tyrosine 3-monooxygenase and other catecholamine-synthesizing enzymes—. Proc. Jpn. Acad. Ser. B.

[58] Eisenhofer G., Kopin I.J., Goldstein D.S. (2004). Catecholamine Metabolism: A Contemporary View with Implications for Physiology and Medicine. Pharmacol. Rev..

[59] Guengerich F.P. (2008). Cytochrome P450 and Chemical Toxicology. Chem. Res. Toxicol..

[60] Nebert D.W., Dalton T.P. (2006). The Role of Cytochrome P450 Enzymes in Endogenous Signalling Pathways and Environmental Carcinogenesis. Nat. Rev. Cancer.

[61] Spickett C.M. (2013). The Lipid Peroxidation Product 4-Hydroxy-2-Nonenal: Advances in Chemistry and Analysis. Redox Biol..

[62] Gago-Dominguez M., Jiang X., Esteban Castelao J. (2007). Lipid Peroxidation and the Protective Effect of Physical Exercise on Breast Cancer. Med. Hypotheses.

[63] Trachootham D., Lu W., Ogasawara M.A., Valle N.R.-D., Huang P. (2008). Redox Regulation of Cell Survival. Antioxid. Redox Signal..

[64] Reuter S., Gupta S.C., Chaturvedi M.M., Aggarwal B.B. (2010). Oxidative Stress, Inflammation, and Cancer: How Are They Linked?. Free Radic. Biol. Med..

[65] Saito J., Onishi N., Yamasaki J., Koike N., Hata Y., Kimura K., Otsuki Y., Nobusue H., Sampetrean O., Shimizu T. (2025). Benzaldehyde Suppresses Epithelial-Mesenchymal Plasticity Overcomes Treatment Resistance in Cancer by Targeting the Interaction of 14-3-3ζ with H3S28ph. Br. J. Cancer.

[66] Lei Y., Cai S., Zhang J.-K., Ding S.-Q., Zhang Z.-H., Zhang C.-D., Dai D.-Q., Li Y.-S. (2025). The Role and Mechanism of Fatty Acid Oxidation in Cancer Drug Resistance. Cell Death Discov..

[67] Furuhashi T., Matsumoto Y., Ishii R., Sugasawa T. (2023). Hypoxia and Lactate Influence VOC Production in A549 Lung Cancer Cells: Mechanisms of Trans 2 Hexenol Formation and Regulation. Front. Mol. Biosci..

[68] Janfaza S., Khorsand B., Nikkhah M., Zahiri J. (2019). Digging Deeper into Volatile Organic Compounds Associated with Cancer. Biol. Methods Protoc..

[69] Zhang Q.-C., Yan W.-L., Jiang L., Zheng Y.-G., Wang J.-X., Zhang R.-K. (2019). Synthesis of Nano-Praseodymium Oxide for Cataluminescence Sensing of Acetophenone in Exhaled Breath. Mol. Basel Switz..

[70] Hershberger C.E., Rodarte A.I., Siddiqi S., Moro A., Acevedo-Moreno L., Brown J.M., Allende D.S., Aucejo F., Rotroff D.M. (2021). Salivary Metabolites Are Promising Non-invasive Biomarkers of Hepatocellular Carcinoma and Chronic Liver Disease. Liver Cancer Int..

[71] Haick H., Broza Y.Y., Mochalski P., Ruzsanyi V., Amann A. (2014). Assessment, Origin, and Implementation of Breath Volatile Cancer Markers. Chem. Soc. Rev..

[72] O’Reilly K.T., Mohler R.E., Zemo D.A., Ahn S., Tiwary A.K., Magaw R.I., Espino Devine C., Synowiec K.A. (2015). Identification of Ester Metabolites from Petroleum Hydrocarbon Biodegradation in Groundwater Using GC×GC-TOFMS. Environ. Toxicol. Chem..

[73] Gandhi N., Das G. (2019). Metabolic Reprogramming in Breast Cancer and Its Therapeutic Implications. Cells.

[74] Lu J., Tan M., Cai Q. (2015). The Warburg Effect in Tumor Progression: Mitochondrial Oxidative Metabolism as an Anti-Metastasis Mechanism. Cancer Lett..

[75] Ley J.P., Engelhart K., Bernhardt J., Bertram H.J. (2002). 3,4-Dihydroxymandelic Acid, a Noradrenalin Metabolite with Powerful Antioxidative Potential. J. Agric. Food Chem..

[76] Miller Fleming L., Olin Sandoval V., Campbell K., Ralser M. (2015). Remaining Mysteries of Molecular Biology: The Role of Polyamines in the Cell. J. Mol. Biol..

[77] Pegg A.E. (2009). Mammalian Polyamine Metabolism and Function. IUBMB Life.

[78] Wißfeld J., Werner A., Yan X., Ten Bosch N., Cui G. (2022). Metabolic Regulation of Immune Responses to Cancer. Cancer Biol. Med..

