# Supplementary material for: Urinary Metabolome Study for Monitoring Prostate Cancer Recurrence Following Radical Prostatectomy
Source: Cancers (Basel). 2025 Aug 24;17(17):2756. doi: 10.3390/cancers17172756 (PMC12427525; doi:10.3390/cancers17172756)
Supplement: Supplementary file 1 [file cancers-17-02756-s001.zip › cancers-3758336-supplementary.pdf]

Supplementary Data

Supplementary Table S1. 155 VOCs that demonstrate significant differences ($p < 0.05$) between the biopsy-designated-positive and biopsy-designated-negative PCa groups generated by PLS-DA.

	VOC (CAS number)	P value
1	001222-05-5	1.97×10^{-21}
2	000088-29-9	4.64×10^{-20}
3	1000458-50-6	1.68×10^{-18}
4	000141-63-9	9.84×10^{-13}
5	002156-97-0	1.43×10^{-12}
6	000541-02-6	1.81×10^{-12}
7	000540-97-6	3.33×10^{-12}
8	000098-86-2	2.66×10^{-11}
9	000124-10-7	5.89×10^{-11}
10	010192-32-2	2.64×10^{-10}
11	1000333-93-9	5.03×10^{-10}
12	043080-23-5	5.91×10^{-10}
13	1000072-26-7	8.96×10^{-10}
14	003076-04-8	1.79×10^{-09}
15	000539-92-4	3.19×10^{-09}
16	1000364-61-2	3.25×10^{-09}
17	000096-76-4	5.21×10^{-09}
18	006386-38-5	6.75×10^{-09}
19	000116-09-6	2.94×10^{-08}
20	015089-22-2	3.47×10^{-08}
21	001120-21-4	4.64×10^{-08}
22	001599-67-3	7.70×10^{-08}
23	000765-70-8	7.96×10^{-08}
24	000927-55-9	1.02×10^{-07}
25	000295-17-0	1.78×10^{-07}
26	000119-61-9	2.20×10^{-07}
27	063493-28-7	2.84×10^{-07}
28	013048-33-4	3.23×10^{-07}
29	000112-41-4	4.53×10^{-07}
30	000124-19-6	6.22×10^{-07}
31	1000366-57-5	6.24×10^{-07}
32	000112-39-0	7.51×10^{-07}
33	000638-66-4	7.54×10^{-07}
34	010493-98-8	1.63×10^{-06}
35	037112-31-5	2.51×10^{-06}
36	001892-22-4	2.83×10^{-06}
37	1000140-77-5	3.05×10^{-06}
38	1000352-81-3	3.59×10^{-06}
39	000617-84-5	4.70×10^{-06}
40	072439-78-2	5.32×10^{-06}
41	017862-85-0	5.66×10^{-06}
42	081671-45-6	5.70×10^{-06}
43	002919-23-5	7.29×10^{-06}
44	001120-36-1	9.74×10^{-06}
45	000064-19-7	1.26×10^{-05}
46	051655-65-3	1.28×10^{-05}
47	097389-69-0	1.48×10^{-05}

48	1000417-00-9	1.53×10^{-05}
49	001192-33-2	1.89×10^{-05}
50	000057-10-3	2.32×10^{-05}
51	000497-23-4	3.21×10^{-05}
52	1000130-87-5	3.29×10^{-05}
53	002516-33-8	3.66×10^{-05}
54	000140-66-9	3.88×10^{-05}
55	000101-86-0	3.94×10^{-05}
56	1000454-30-7	4.05×10^{-05}
57	001630-94-0	4.10×10^{-05}
58	000616-42-2	4.23×10^{-05}
59	031897-93-5	4.83×10^{-05}
60	1000131-18-9	5.86×10^{-05}
61	1000163-97-9	6.22×10^{-05}
62	036685-48-0	6.28×10^{-05}
63	021391-99-1	7.22×10^{-05}
64	1000306-28-2	8.09×10^{-05}
65	013126-39-1	8.45×10^{-05}
66	000142-50-7	9.49×10^{-05}
67	002136-72-3	9.74×10^{-05}
68	000542-28-9	1.1×10^{-04}
69	000097-63-2	1.12×10^{-04}
70	1000441-73-7	1.16×10^{-04}
71	007206-17-9	1.17×10^{-04}
72	000294-62-2	1.23×10^{-04}
73	1000098-14-8	1.23×10^{-04}
74	093103-70-9	1.24×10^{-04}
75	000883-93-2	1.25×10^{-04}
76	004389-50-8	1.30×10^{-04}
77	000629-50-5	1.47×10^{-04}
78	002305-36-4	1.56×10^{-04}
79	1000416-85-2	1.64×10^{-04}
80	000872-05-9	1.74×10^{-04}
81	053907-81-6	2.00×10^{-04}
82	006175-49-1	2.03×10^{-04}
83	000295-02-3	2.08×10^{-04}
84	1000406-32-4	2.08×10^{-04}
85	040710-32-5	2.18×10^{-04}
86	000098-00-0	2.33×10^{-04}
87	000544-76-3	2.67×10^{-04}
88	000101-39-3	2.74×10^{-04}
89	1000400-52-5	2.81×10^{-04}
90	1000420-86-0	2.95×10^{-04}
91	000128-09-6	3.06×10^{-04}
92	000096-48-0	3.09×10^{-04}
93	003524-73-0	3.19×10^{-04}
94	1000421-67-0	3.24×10^{-04}
95	1000222-86-6	3.25×10^{-04}
96	013674-84-5	3.28×10^{-04}
97	007390-81-0	3.73×10^{-04}
98	1000357-25-8	3.85×10^{-04}
99	1000421-16-2	3.91×10^{-04}
100	055282-12-7	4.12×10^{-04}
101	055449-66-6	4.35×10^{-04}

102	1000420-85-9	4.36×10^{-04}
103	050442-70-1	4.73×10^{-04}
104	000112-31-2	5.63×10^{-04}
105	1000406-34-9	6.11×10^{-04}
106	018919-94-3	6.65×10^{-04}
107	002039-76-1	6.84×10^{-04}
108	000088-58-4	6.95×10^{-04}
109	000556-71-8	7.63×10^{-04}
110	1000375-02-1	7.71×10^{-04}
111	1000382-54-4	7.93×10^{-04}
112	041446-65-5	8.08×10^{-04}
113	1000146-59-1	8.36×10^{-04}
114	002425-77-6	9.11×10^{-03}
115	139123-69-6	1.03×10^{-03}
116	000104-76-7	1.03×10^{-03}
117	006418-47-9	1.06×10^{-03}
118	002941-78-8	1.10×10^{-03}
119	000693-71-0	1.11×10^{-03}
120	056114-62-6	1.11×10^{-03}
121	007206-15-7	1.17×10^{-03}
122	013360-61-7	1.18×10^{-03}
123	002437-56-1	1.26×10^{-03}
124	007206-16-8	1.29×10^{-03}
125	006846-50-0	1.29×10^{-03}
126	1000299-33-6	1.31×10^{-03}
127	1000297-17-8	1.35×10^{-03}
128	1000259-58-5	1.40×10^{-03}
129	000593-49-7	1.43×10^{-03}
130	000629-73-2	1.44×10^{-03}
131	1000130-84-8	1.44×10^{-03}
132	025237-79-0	1.45×10^{-03}
133	1000465-65-6	1.47×10^{-03}
134	000302-72-7	1.47×10^{-03}
135	1000334-84-1	1.49×10^{-03}
136	028715-26-6	1.51×10^{-03}
137	000605-67-4	1.58×10^{-03}
138	006938-94-9	1.62×10^{-03}
139	001569-59-1	1.67×10^{-03}
140	003055-93-4	1.68×10^{-03}
141	037148-65-5	1.69×10^{-03}
142	000100-42-5	1.76×10^{-03}
143	028336-57-4	1.80×10^{-03}
144	004443-61-2	1.84×10^{-03}
145	002847-72-5	1.85×10^{-03}
146	000608-43-5	1.88×10^{-03}
147	092300-81-7	1.90×10^{-03}
148	1000417-01-0	1.94×10^{-03}
149	007206-26-0	2.04×10^{-03}
150	1000406-34-3	2.05×10^{-03}
151	000065-85-0	2.09×10^{-03}
152	000084-74-2	2.25×10^{-03}
153	002305-05-7	2.31×10^{-03}
154	000638-67-5	2.34×10^{-03}
155	028732-75-4	2.37×10^{-03}

Supplementary Table S2. 157 VOCs that demonstrate significant differences ($p < 0.05$) between the pre- and post-RP groups generated by PLS-DA.

	VOC (CAS number)	P value	Expression Response in Post Surgery
1	000098-86-2	1.48×10^{-26}	Down
2	001222-05-5	5.34×10^{-15}	Down
3	000541-02-6	$5.96 \text{ X } 10^{-15}$	Down
4	000088-29-9	6.74×10^{-15}	Down
5	000540-97-6	1.32×10^{-13}	Down
6	000556-52-5	5.79×10^{-12}	Down
7	018919-94-3	1.69×10^{-11}	Down
8	000927-55-9	1.50×10^{-10}	Down
9	001892-22-4	2.98×10^{-10}	Down
10	000141-62-8	9.80×10^{-10}	Down
11	017862-85-0	1.10×10^{-09}	Down
12	015089-22-2	1.21×10^{-09}	Down
13	000124-10-7	4.21×10^{-09}	Down
14	000295-17-0	5.67×10^{-09}	Down
15	000096-76-4	6.38×10^{-09}	Down
16	000541-01-5	7.19×10^{-09}	Down
17	024569-83-3	7.42×10^{-09}	Down
18	000638-66-4	1.10×10^{-08}	Down
19	037112-31-5	1.19×10^{-08}	Down
20	007045-71-8	1.46×10^{-08}	Up
21	006386-38-5	1.76×10^{-08}	Down
22	001120-21-4	2.87×10^{-08}	Down
23	000112-41-4	2.92×10^{-08}	Down
24	061141-72-8	5.91×10^{-08}	Up
25	1000098-14-8	5.92×10^{-08}	Down
26	017302-23-7	7.13×10^{-08}	Up
27	013151-34-3	7.38×10^{-08}	Up
28	000057-10-3	1.05×10^{-07}	Down
29	001120-36-1	2.27×10^{-07}	Down
30	1000306-28-2	2.92×10^{-07}	Down
31	000119-61-9	3.47×10^{-07}	Down
32	000112-40-3	6.24×10^{-07}	Up
33	000872-05-9	6.41×10^{-07}	Down
34	000112-39-0	6.60×10^{-07}	Down
35	1000458-50-6	9.36×10^{-07}	Down
36	006117-97-1	1.10×10^{-06}	Up
37	026730-12-1	2.05×10^{-06}	Up
38	062016-18-6	2.19×10^{-06}	Up
39	000141-63-9	2.55×10^{-06}	Down
40	013048-33-4	2.95×10^{-06}	Down
41	002847-72-5	4.04×10^{-06}	Up
42	000629-59-4	4.10×10^{-06}	Up

43	002980-69-0	4.39×10^{-06}	Up
44	006175-49-1	4.57×10^{-06}	Down
45	000542-28-9	4.59×10^{-06}	Down
46	019218-94-1	6.12×10^{-06}	Up
47	006846-50-0	8.27×10^{-06}	Down
48	000126-86-3	9.44×10^{-06}	Down
49	1000364-61-2	1.01×10^{-05}	Down
50	017301-23-4	1.08×10^{-05}	Up
51	103439-07-2	1.50×10^{-05}	Down
52	003737-95-9	1.57×10^{-05}	Up
53	000124-19-6	1.66×10^{-05}	Down
54	041446-65-5	1.78×10^{-05}	Down
55	000116-09-6	1.81×10^{-05}	Down
56	010493-98-8	1.98×10^{-05}	Down
57	001636-44-8	2.48×10^{-05}	Up
58	025117-26-4	2.65×10^{-05}	Up
59	017301-22-3	2.75×10^{-05}	Up
60	000105-41-9	2.96×10^{-05}	Up
61	001454-84-8	3.36×10^{-05}	Down
62	000104-76-7	3.81×10^{-05}	Down
63	028715-26-6	3.81×10^{-05}	Down
64	002213-23-2	3.86×10^{-05}	Up
65	068595-69-7	4.15×10^{-05}	Down
66	071525-41-2	4.19×10^{-05}	Up
67	000765-70-8	4.37×10^{-05}	Down
68	001630-94-0	4.47×10^{-05}	Down
69	000101-39-3	4.66×10^{-05}	Down
70	000112-61-8	5.37×10^{-05}	Down
71	013287-21-3	5.71×10^{-05}	Up
72	000497-23-4	6.06×10^{-05}	Down
73	1000366-57-5	7.06×10^{-05}	Down
74	000556-71-8	7.37×10^{-05}	Down
75	000057-56-7	7.56×10^{-05}	Down
76	1000417-00-9	7.59×10^{-05}	Down
77	000057-11-4	8.71×10^{-05}	Down
78	056114-62-6	8.96×10^{-05}	Down
79	000588-06-7	9.07×10^{-05}	Down
80	000539-92-4	9.40×10^{-05}	Down
81	017302-27-1	1.07×10^{-04}	Up
82	1000406-35-0	1.08×10^{-04}	Up
83	007206-16-8	1.15×10^{-04}	Down
84	017312-54-8	1.30×10^{-04}	Up
85	1000156-09-4	1.32×10^{-04}	Down
86	004292-19-7	1.33×10^{-04}	Up
87	000617-84-5	1.48×10^{-04}	Down
88	002156-97-0	1.52×10^{-04}	Down
89	038147-00-1	1.57×10^{-04}	Down
90	019095-24-0	1.62×10^{-04}	Down
91	1000406-31-8	1.67×10^{-04}	Up

92	000075-31-0	1.67×10^{-04}	Up
93	074036-95-6	1.76×10^{-04}	Up
94	000112-31-2	1.98×10^{-04}	Down
95	031701-78-7	2.48×10^{-04}	Up
96	054211-16-4	2.82×10^{-04}	Down
97	000621-42-1	3.17×10^{-04}	Down
98	062108-27-4	3.28×10^{-04}	Up
99	188907-99-5	3.54×10^{-04}	Down
100	000112-88-9	3.54×10^{-04}	Down
101	002529-64-8	4.23×10^{-04}	Down
102	071579-69-6	4.58×10^{-04}	Down
103	017301-28-9	4.60×10^{-04}	Down
104	019095-23-9	5.57×10^{-04}	Up
105	1000146-59-1	5.65×10^{-04}	Down
106	031295-56-4	5.95×10^{-04}	Up
107	1000072-26-7	6.41×10^{-04}	Down
108	002078-13-9	6.59×10^{-04}	Down
109	019424-26-1	6.61×10^{-04}	Up
110	000097-63-2	6.96×10^{-04}	Down
111	1000382-90-8	7.21×10^{-04}	Up
112	1000417-01-0	7.29×10^{-04}	Down
113	021391-99-1	7.57×10^{-04}	Down
114	043080-23-5	8.17×10^{-04}	Down
115	003076-04-8	8.67×10^{-04}	Down
116	000593-45-3	8.69×10^{-04}	Down
117	1000461-70-8	8.74×10^{-04}	Down
118	1000347-25-6	9.29×10^{-04}	Down
119	1000309-34-3	9.59×10^{-04}	Up
120	1000352-81-3	9.93×10^{-04}	Down
121	007225-66-3	9.98×10^{-04}	Up
122	007390-81-0	1.00×10^{-03}	Down
123	136091-23-1	1.02×10^{-03}	Up
124	035599-77-0	1.03×10^{-03}	Up
125	000064-19-7	1.07×10^{-03}	Down
126	075163-97-2	1.08×10^{-03}	Up
127	002402-06-4	1.12×10^{-03}	Down
128	000498-40-8	1.23×10^{-03}	Up
129	021964-49-8	1.34×10^{-03}	Down
130	081671-45-6	1.45×10^{-03}	Down
131	000629-73-2	1.52×10^{-03}	Down
132	013360-61-7	1.59×10^{-03}	Down
133	000926-39-6	1.61×10^{-03}	Up
134	1000256-39-2	1.62×10^{-03}	Up
135	002437-56-1	1.63×10^{-03}	Down
136	063493-28-7	1.78×10^{-03}	Up
137	1000395-75-6	1.80×10^{-03}	Down
138	001691-13-0	1.89×10^{-03}	Up
139	000822-67-3	2.01×10^{-03}	Down
140	000112-54-9	2.17×10^{-03}	Down

141	1000406-04-4	2.21×10^{-03}	Up
142	026741-18-4	2.21×10^{-03}	Up
143	075163-98-3	2.23×10^{-03}	Up
144	1000309-16-3	2.28×10^{-03}	Up
145	000108-94-1	2.29×10^{-03}	Down
146	001477-63-0	2.34×10^{-03}	Down
147	1000441-73-7	2.36×10^{-03}	Down
148	1000316-17-5	2.39×10^{-03}	Down
149	031613-73-7	2.46×10^{-03}	Down
150	002425-77-6	2.51×10^{-03}	Down
151	041678-32-4	2.52×10^{-03}	Down
152	013897-37-5	2.57×10^{-03}	Down
153	055282-12-7	2.90×10^{-03}	Down
154	007206-28-2	2.99×10^{-03}	Down
155	000629-50-5	3.02×10^{-03}	Down
156	729602-94-2	3.14×10^{-03}	Down
157	000921-47-1	3.30×10^{-03}	Up

Supplementary Table S3. The Compounds Names of the VOCs included in Figures 1–5.

	CAS #	Chemical Name	Chemical Formula	Higher in
	Figure 1B			
1	1000458-50-6	1,3,5,7,9-Pentasiloxane, 1,1,3,3,5,5,7,7,9,9-decamethyl-1,9-di(tert.butyl)-	$C_{18}H_{44}O_4Si_5$	PCa +
2	1222-05-5	Cyclopenta[g]-2-benzopyran, 1,3,4,6,7,8-hexahydro-4,6,6,7,8,8-hexamethyl-	$C_{18}H_{26}O$	PCa +
3	88-29-9	7-Acetyl-6-ethyl-1,1,4,4-tetramethyltetralin	$C_{18}H_{26}O$	PCa +
4	141-63-9	Pentasiloxane, dodecamethyl-	$C_{12}H_{36}O_4Si5$	PCa +
5	2156-97-0	Dodecyl acrylate	$C_{15}H_{28}O_2$	PCa +
6	1000333-93-9			PCa +
7	43080-23-5	2-Propenoic acid, pentadecyl ester	$C_{18}H_{34}O_2$	PCa +
8	3076-04-8	2-Propenoic acid, tridecyl ester	$C_{16}H_{30}O_2$	PCa +
9	15089-22-2	N-Benzyl-N-ethyl-p-isopropylbenzamide	$C_{19}H_{23}NO$	PCa +
10	539-92-4	Carbonic acid, bis(2-methylpropyl) ester	$C_9H_{18}O_3$	PCa +
11	124-10-7	Methyl tetradecanoate	$C_{15}H_{30}O_2$	PCa +
12	1000072-26-7	N-(Trifluoroacetyl)-N,O,O',O''-tetrakis(trimethylsilyl)norepinephrine	$C_{22}H_{42}F_3NO_4Si_4$	PCa +
13	765-70-8	1,2-Cyclopentanedione, 3-methyl-	$C_6H_8O_2$	PCa +
14	116-09-6	2-Propanone, 1-hydroxy-	$C_3H_6O_2$	PCa +
15	98-86-2	Acetophenone	C_8H_8O	PCa +
16	64-19-7	Acetic acid	$C_2H_4O_2$	PCa +
17	1000352-81-3	2,6-Dihydroxyacetophenone, 2TMS derivative	$C_{14}H_{24}O_3Si_2$	PCa +
18	1000366-57-5	Tris(tert-butyldimethylsilyloxy)arsane	$C_{18}H_{45}AsO_3Si_3$	PCa +
19	927-55-9	1-Pentanol, 4-amino-	$C_5H_{13}NO$	PCa +
20	295-17-0	Cyclotetradecane	$C_{14}H_{28}$	PCa +
21	638-66-4	Octadecanal	$C_{18}H_{36}O$	PCa +
22	4389-50-8	2-Amino-6-methylbenzoic acid	$C_8H_9NO_2$	PCa +
23	1000364-61-2	1,1,3,3,5,5,7,7-Octamethyl-7-(2-methylpropoxy)tetrasiloxan-1-ol	$C_{12}H_{34}O_5Si_4$	PCa +
24	2919-23-5	Cyclobutanol	C_4H_7OH	PCa +
25	10493-98-8	2-Cyclopenten-1-one, 2-hydroxy-	$C_5H_6O_2$	PCa +
26	1599-67-3	1-Docosene	$C_{22}H_{44}$	PCa -
27	1000140-77-5	Pentanoic acid, 2,2,4-trimethyl-3-carboxyisopropyl, isobutyl ester	$C_{16}H_{30}O_4$	PCa +
28	97389-69-0	2'-Hydroxy-5'-methylacetophenone, TMS derivative	$C_{12}H_{18}O_3Si$	PCa -
29	17862-85-0	3-Methoxyamphetamine	$C_{10}H_{15}NO$	PCa +
30	37112-31-5	Levoglucosenone	$C_6H_6O_3$	PCa +
	Figure 2			
1	1222-05-5	Cyclopenta[g]-2-benzopyran, 1,3,4,6,7,8-hexahydro-4,6,6,7,8,8-hexamethyl-	$C_{18}H_{26}O$	
2	88-29-9	7-Acetyl-6-ethyl-1,1,4,4-tetramethyltetralin	$C_{18}H_{26}O$	
3	1000458-50-6	1,3,5,7,9-Pentasiloxane, 1,1,3,3,5,5,7,7,9,9-decamethyl-1,9-di(tert.butyl)-	$C_{18}H_{44}O_4Si_5$	
4	141-63-9	Pentasiloxane, dodecamethyl-	$C_{12}H_{36}O_4Si_5$	
5	2156-97-0	Dodecyl acrylate	$C_{15}H_{28}O_2$	

6	97389-69-0	2'-Hydroxy-5'-methylacetophenone, TMS derivative	$C_{12}H_{18}O_3Si$	
7	1599-67-3	1-Docosene	$C_{22}H_{44}$	
8	93103-70-9	2-(Acetoxymethyl)-3-(methoxycarbonyl)biphenylene	$C_{17}H_{14}O_4$	
	Figure 3B			
1	98-86-2	Acetophenone	C_8H_8O	pre
2	1222-05-5	Cyclopenta[g]-2-benzopyran, 1,3,4,6,7,8-hexahydro-4,6,6,7,8,8-hexamethyl-	$C_{18}H_{26}O$	pre
3	88-29-9	7-Acetyl-6-ethyl-1,1,4,4-tetramethyltetralin	$C_{18}H_{26}O$	pre
4	15089-22-2	N-Benzyl-N-ethyl-p-isopropylbenzamide	$C_{19}H_{23}NO$	pre
5	556-52-5	Glycidol	$C_3H_6O_2$	pre
6	24569-83-3	Acetic acid, 2-(N-methyl-N-phosphonatomethyl)amino-	$C_4H_{10}NO_5P$	pre
7	927-55-9	1-Pentanol, 4-amino-	$C_5H_{13}NO$	pre
8	141-62-8	Tetrasiloxane, decamethyl-	$C_{10}H_{30}O_3Si_4$	pre
9	17862-85-0	3-Methoxyamphetamine	$C_{10}H_{15}NO$	pre
10	638-66-4	Octadecanal	$C_{18}H_{36}O$	pre
11	541-01-5	Heptasiloxane, hexadecamethyl-	$C_{16}H_{48}O_6Si_7$	pre
12	37112-31-5	Levoglucosenone	$C_6H_6O_3$	pre
13	1000458-50-6	1,3,5,7,9-Pentasiloxane, 1,1,3,3,5,5,7,7,9,9-decamethyl-1,9-di(tert.butyl)-	$C_{18}H_{44}O_4Si_5$	pre
14	141-63-9	Pentasiloxane, dodecamethyl-	$C_{12}H_{36}O_4Si_5$	pre
15	1892-22-4	3-Aminopiperidin-2-one	$C_5H_{10}N_2O$	pre
16	17302-23-7	Nonane, 4,5-dimethyl-	$C_{11}H_{24}$	post
17	295-17-0	Cyclotetradecane	$C_{14}H_{28}$	pre
18	124-10-7	Methyl tetradecanoate	$C_{15}H_{30}O_2$	pre
19	7045-71-8	Undecane, 2-methyl-	$C_{12}H_{26}$	post
20	18919-94-3	Tetracosamethyl-cyclododecasiloxane	$C_{24}H_{72}O_{12}Si_{12}$	pre
21	112-40-3	Dodecane	$C_{12}H_{26}$	post
22	1000306-28-2	Tris(trimethylsilyl)-(p-methoxybenzoyl)silane	$C_{17}H_{34}O_2Si_4$	pre
23	1000098-14-8	1,4:3,6-Dianhydro-.alpha.-d-glucopyranose	$C_6H_8O_4$	pre
24	41446-65-5	4-Tetradecene, (Z)-	$C_{14}H_{28}$	pre
25	765-70-8	1,2-Cyclopentanedione, 3-methyl-	$C_6H_8O_2$	pre
	Figure 4B			
1	15089-22-2	N-Benzyl-N-ethyl-p-isopropylbenzamide	$C_{19}H_{23}NO$	low in healthy
2	141-63-9	Pentasiloxane, dodecamethyl-	$C_{12}H_{36}O_4Si_5$	low in healthy
3	295-17-0	Cyclotetradecane	$C_{14}H_{28}$	low in healthy
4	126-86-3	2,4,7,9-Tetramethyl-5-decyn-4,7-diol	$C_{14}H_{26}O_2$	low in healthy
5	765-70-8	1,2-Cyclopentanedione, 3-methyl-	$C_6H_8O_2$	low in healthy
6	124-10-7	Methyl tetradecanoate	$C_{15}H_{30}O_2$	low in healthy
7	41446-65-5	4-Tetradecene, (Z)-	$C_{14}H_{28}$	lowest in RCH

8	1000458-50-6	1,3,5,7,9-Pentasiloxane, 1,1,3,3,5,5,7,7,9,9-decamethyl-1,9-di(tert.butyl)-	$C_{18}H_{44}O_4Si_5$	lowest in RCH
9	995-82-4	Hexasiloxane, 1,1,3,3,5,5,7,7,9,9,11,11-dodecamethyl-	$C_{12}H_{36}O_5Si_6$	lowest in RCH
10	112-40-3	Dodecane	$C_{12}H_{26}$	lowest in RCH
11	55282-12-7	Octadecane, 3-ethyl-5-(2-ethylbutyl)-	$C_{26}H_{54}$	lowest in RCH
12	116-09-6	2-Propanone, 1-hydroxy-	$C_3H_6O_2$	lowest in RCH
13	1222-05-5	Cyclopenta[g]-2-benzopyran, 1,3,4,6,7,8-hexahydro-4,6,6,7,8,8-hexamethyl-	$C_{18}H_{26}O$	lowest in RCH
14	141-62-8	Tetrasiloxane, decamethyl-	$C_{10}H_{30}O_3Si_4$	lowest in RCH
15	88-29-9	7-Acetyl-6-ethyl-1,1,4,4-tetramethyltetralin	$C_{18}H_{26}O$	lowest in RCH
16	112-31-2	Decanal	$C_{10}H_{20}O$	lowest in RCH
17	53907-81-6	2-Heptanamine, 5-methyl-	$C_8H_{19}N$	lowest in RCH
18	1000406-34-3	Eicosyl isopropyl ether	$C_{23}H_{48}O$	lowest in RCH
19	7206-28-2	5-Dodecene, (Z)-	$C_{12}H_{24}$	lowest in RCH
20	98-86-2	Acetophenone	C_8H_8O	lowest in RCH
	Figure 4D			
1	53907-81-6	2-Heptanamine, 5-methyl-	$C_8H_{19}N$	lowest in RCH
2	126-86-3	2,4,7,9-Tetramethyl-5-decyn-4,7-diol	$C_{14}H_{26}O_2$	lowest in RCH
3	1502-38-1	Cyclooctane, methyl-	C_9H_{18}	lowest in RCH
4	84-74-2	Dibutyl phthalate	$C_{16}H_{22}O_4$	lowest in RCH
5	141-63-9	Pentasiloxane, dodecamethyl-	$C_{12}H_{36}O_4Si_5$	lowest in RCH
6	7206-28-2	5-Dodecene, (Z)-	$C_{12}H_{24}$	lowest in RCH
7	765-70-8	1,2-Cyclopentanedione, 3-methyl-	$C_6H_8O_2$	lowest in RCH
8	1000383-11-5	Carbonic acid, octadecyl prop-1-en-2-yl ester	$C_{22}H_{42}O_3$	lowest in RCH
9	15089-22-2	N-Benzyl-N-ethyl-p-isopropylbenzamide	$C_{19}H_{23}NO$	lowest in RCH
10	1000421-54-9	Silane, methylvinyl(2-methylpent-3-yloxy)(methylvinyldodecyloxysilyloxy)-	$C_{24}H_{50}O_3Si_2$	lowest in RCH
11	295-17-0	Cyclotetradecane	$C_{14}H_{28}$	lowest in RCH

12	1000351-80-0	Triacontyl pentafluoropropionate	$C_{33}H_{61}F_5O_2$	lowest in RCH
13	36653-82-4	1-Hexadecanol	$C_{16}H_{34}O$	lowest in RCH
14	1000406-34-3	Eicosyl isopropyl ether	$C_{23}H_{48}O$	lowest in RCH
15	1000210-65-7	1-Hydroxymethyl-7,7-dimethyl-bicyclo[2.2.1]heptane-2,3-dione	$C_{10}H_{14}O_3$	lowest in RCH
16	124-10-7	Methyl tetradecanoate	$C_{15}H_{30}O_2$	lowest in RCH
17	372-20-3	3-Fluorophenol	FC_6H_5O	lowest in RCH
18	1000309-38-2	Oxalic acid, isobutyl heptadecyl ester	$C_{23}H_{44}O_4$	lowest in RCH
19	502-69-2	2-Pentadecanone, 6,10,14-trimethyl-	$C_{18}H_{36}O$	lowest in RCH
20	995-82-4	Hexasiloxane, 1,1,3,3,5,5,7,7,9,9,11,11-dodecamethyl-	$C_{12}H_{36}O_5Si_6$	lowest in RCH
21	195194-80-0	2-Piperidinone, N-[4-bromo-n-butyl]-	$C_9H_{16}BrNO$	lowest in RCH
22	3386-33-2	Octadecane, 1-chloro-	$C_{18}H_{37}Cl$	lowest in RCH
	Figure 4F			
1	535-77-3	Benzene, 1-methyl-3-(1-methylethyl)-	$C_{10}H_{14}$	BCR
2	1540-80-3	1,8-Cyclotetradecadiyne	$C_{14}H_{20}$	BCR
3	99-87-6	p-Cymene	$C_{10}H_{14}$	BCR
4	3386-33-2	Octadecane, 1-chloro-	$C_{18}H_{37}Cl$	RCM
5	71579-69-6	3-Isopropoxy-1,1,1,7,7,7-hexamethyl-3,5,5-tris(trimethylsiloxy)tetrasiloxane	$C_{18}H_{52}O_7Si_7$	BCR
6	6175-49-1	2-Dodecanone	$C_{12}H_{24}O$	BCR
7	883-93-2	Benzothiazole, 2-phenyl-	$C_{13}H_9NS$	RCM
8	4784-86-5	1,3-Cyclopentadiene, 1,2-dimethyl-	C_7H_{10}	BCR
9	1000388-83-8	5-Methoxy-2-methyl-9-oxa-1-azatetracyclo[8.7.0.0(3,8).0(11,16)]heptadeca-3(8),4,6,11(16),12,14-hexaen-17-one	$C_{17}H_{15}NO_3$	BCR
10	37148-65-5	3,4-Dihydroxymandelic acid, 4TMS derivative	$C_{20}H_{40}O_5Si_4$	BCR
11	1000408-12-9	2-{[(Trimethylsilyl)oxy]carbonyl}phenyl 2-[(trimethylsilyl)oxy]benzoate	$C_{19}H_{26}O_5Si_2$	BCR
12	1000405-65-6	Fumaric acid, 2-methylpentyl tridec-2-yn-1-yl ester	$C_{23}H_{38}O_4$	BCR
13	2345-27-9	2-Tetradecanone	$C_{14}H_{28}O$	BCR
14	107-68-6	N-Methyltaurine	$C_3H_9NO_3S$	BCR
15	6443-92-1	(Z)-2-Heptene	C_7H_{14}	BCR
16	918-05-8	Methanesulfonamide, N,N-dimethyl-	$C_3H_9NO_2S$	BCR
17	1000309-16-4	Sulfurous acid, pentadecyl 2-pentyl ester	$C_{20}H_{42}O_3S$	BCR
18	1000336-52-6	Octadecane-1,2-diol, 2TMS derivative	$C_{24}H_{54}O_2Si_2$	BCR
19	1000268-80-8	Pyrazol-5(4H)-one, 1-acetyl-4-allyl-3-methyl-	$C_9H_{12}N_2O_2$	BCR
20	1686-20-0	p-Mentha-1,5-dien-8-ol	$C_{10}H_{16}O$	BCR
	Figure 5			

1	98-86-2	Acetophenone	C_8H_8O
2	1222-05-5	Cyclopenta[g]-2-benzopyran, 1,3,4,6,7,8-hexahydro-4,6,6,7,8,8-hexamethyl-	$C_{18}H_{26}O$
3	927-55-9	1-Pentanol, 4-amino-	$C_5H_{13}NO$
4	2529-64-8	17beta-Estradiol, 3-deoxy-	$C_{18}H_{24}O$
5	88-29-9	7-Acetyl-6-ethyl-1,1,4,4-tetramethyltetralin	$C_{18}H_{26}O$
6	541-02-6	Decamethylcyclopentasiloxane	$C_{10}H_{30}O_5Si_5$
7	295-17-0	Cyclotetradecane	$C_{14}H_{28}$
8	541-01-5	HEXADECAMETHYLHEPTASILOXANE	$C_{16}H_{48}O_6Si_7$
